# Resting Rates of Blood Flow and Glucose Use per Neuron Are Proportional to Number of Endothelial Cells Available per Neuron Across Sites in the Rat Brain

**DOI:** 10.3389/fnint.2022.821850

**Published:** 2022-06-10

**Authors:** Lissa Ventura-Antunes, Oisharya Moon Dasgupta, Suzana Herculano-Houzel

**Affiliations:** ^1^Department of Psychology, Vanderbilt University, Nashville, TN, United States; ^2^Department of Biological Sciences, Vanderbilt University, Nashville, TN, United States; ^3^Vanderbilt Brain Institute, Vanderbilt University, Nashville, TN, United States

**Keywords:** metabolism, capillary density, brain vasculature, brain energetics, neuronal density

## Abstract

We report in a companion paper that in the mouse brain, in contrast to the 1,000-fold variation in local neuronal densities across sites, capillary density (measured both as capillary volume fraction and as density of endothelial cells) show very little variation, of the order of only fourfold. Here we confirm that finding in the rat brain and, using published rates of local blood flow and glucose use at rest, proceed to show that what small variation exists in capillary density across sites in the rat brain is strongly and linearly correlated to variations in local rates of brain metabolism at rest. Crucially, we show that such variations in local capillary density and brain metabolism are not correlated with local variations in neuronal density, which contradicts expectations that use-dependent self-organization would cause brain sites with more neurons to have higher capillary densities due to higher energetic demands. In fact, we show that the ratio of endothelial cells per neuron serves as a linear indicator of average blood flow and glucose use per neuron at rest, and both increase as neuronal density decreases across sites. In other words, because of the relatively tiny variation in capillary densities compared to the large variation in neuronal densities, the anatomical infrastructure of the brain is such that those sites with fewer neurons have more energy supplied per neuron, which matches a higher average rate of energy use per neuron, compared to sites with more neurons. Taken together, our data support the interpretation that resting brain metabolism is not demand-based, but rather limited by its capillary supply, and raise multiple implications for the differential vulnerability of diverse brain areas to disease and aging.

## Introduction

The brain, which together with the liver is the most energy-consuming organ in the human body, maintains its functionality through a nearly constant supply of energy through its circulatory system (Aschoff et al., 1971; Mintun et al., 2002), even as variations in neuronal activity cause local changes in rate of blood flow and ensuing rates of energy use (Kety, 1956; Iadecola, 2017). While such local changes are consistent with the dominant demand-based view of brain metabolism, according to which the increasing requirements for energy posed by increasing levels of neuronal activity are met by increased energy supply (based on the seminal findings of Attwell and Laughlin (2001)), the demand-based framework fails to explain how come local increases in rates of energy supply and use do not translate into overall, brain-wide increases in energy supply and use. Instead, local increases in neuronal firing activity and ensuing rates of energy supply and use are always accompanied by decreases elsewhere in the brain, such that overall, the blood supply and energy economy of the brain as a whole remains stable over time (Mintun et al., 2002).

Such a trade-off in activity across brain sites would be possible if there were a third party governing energy demands and allocation at all times, one that decreases energy allocation to one site as it increases energy allocation to another. Such a scenario seems unlikely in the highly distributed system that is the mammalian brain.

A much simpler alternative is that blood circulation in the brain works as a closed system, as it does in the body as a whole, with no variation in circulating blood volume, such that increased circulation to one part happens at the expense of decreased circulation to another part. The simplest scenario in which the brain would operate as one such closed system, with limited blood resources in the form of a constant rate of blood flow, is one where the brain functions at near capacity already at “rest” (Herculano-Houzel and Rothman, 2022). The anatomical circumstances that would allow near-capacity function at rest would occur in any organ whose capillaries were always patent – unlike in skeletal muscle, the gastrointestinal tract, or the penis, for example, where massive capillary recruitment parallels variations in activity (Shepherd, 1982; Andersson, 2011; Skattebo et al., 2020). Indeed, the brain is one such organ with a constitutively patent capillary bed, where variations in sleep-waking states and in levels of waking activity occur with hardly any significant capillary recruitment (Kuschinsky and Paulson, 1992; Zoccoli et al., 1996; Jesperson and Ostergaard, 2012).

We propose in an accompanying manuscript a new framework of brain metabolism that views energy use in the brain as constitutively near maximum and constrained by blood supply to brain tissue, whose rate is ultimately limited by the density of capillaries in the brain (Krogh, 1919; Gjedde, 2005; Herculano-Houzel and Rothman, 2022). In a previous study, we found that capillary densities in the mouse brain are not variable in coordination with neuronal densities across sites, a finding that refutes a basic expectation of the demand-based view of brain metabolism^1^, but only supports a supply-limited framework if local capillary densities are found to correlate strongly, and linearly, with local rates of blood flow. Moreover, while the demand-based view of brain metabolism predicts local rates of blood flow and energy use to correlate with neuronal densities, our supply-limited view predicts independence between neuronal local densities and metabolic rates.

Previous studies showed that local capillary densities indeed correlate strongly, and linearly, with local rates of blood flow, with highest rates and densities in the inferior colliculus and lowest in the white matter (Klein et al., 1986; Borowsky and Collins, 1989). Here we determine capillary densities (measured both as capillary volume fraction and as endothelial cell densities) in different anatomically identified areas of the rat brain and compare those densities to reported measurements of local blood flow and rate of glucose use in matching areas. We find that local capillary densities, but not neuronal densities, are indeed a strong and linear predictor of local rates of blood flow and glucose use in the resting rat brain, meeting the necessary condition for a supply-limited framework of brain metabolism.

## Materials and Methods

### Experimental Design

Our goal with this study was simply to determine whether local variations in capillary density across sites in the rat brain correlate closely with local variations in rate of glucose use at rest reported in the literature. To this end, therefore, only cell densities and capillary density (measured as capillary volume fraction) are required, not absolute quantities. We used structured illumination confocal imaging under high magnification to allow quantification of cell and capillary densities in thin optical sections of multiple structures in the brain of three adult Sprague-Dawley rats. This approach is based on our finding in an accompanying paper that 2D and 3D quantifications of cellular density and capillary fraction give equivalent results, with similarly strong relationships between capillary density and capillary fraction, and across densities of the different cell types (Ventura-Antunes and Herculano-Houzel, in review), and leverages the efficiency of using 2D analysis with the high resolution and thin optical section provided by high magnification with a 63x objective in confocal imaging. Structures were chosen to match locations, listed in Table 1, with measured local rates of glucose use and/or blood flow at rest reported in the literature (Sokoloff et al., 1977; McCulloch et al., 1982; Nakai et al., 1983; Gjedde and Diemer, 1985; Levant and Pazdernik, 2004). These reports in the literature, in turn, were selected for their use of similar methods, and for their reasonably extensive analysis of multiple, and overlapping, brain sites, necessary for the present analysis. Brain capillaries were revealed by immunohistochemistry against collagen IV, which labels the basal lamina of blood vessels. Immunohistochemistry against the neuronal marker NeuN (Mullen et al., 1992) was used to reveal neurons; glial cells were identified by exclusion of neurons and capillary-associated cells from the total number of cell nuclei visualized with DAPI. In each location, we quantified capillary area fraction (which is identical to volume fraction), densities of neurons, glial, and capillary-associated (heretofore “endothelial”) cells. Analysis consisted of determining whether those variables were correlated with reported rates of glucose use at rest across locations and animals.

**TABLE 1:** Relative cellular composition of different structures of the rat brain.

Structure	*n*	%Vascular fraction	%Endothelial cells	%Neuronal cells	%Glial cells	%Glial cell of all non-neuronal cells	% Endothelial cells of all non-N
Hypothalamus	27	3.22 ± 1.09%	13.13 ± 3.36%	42.36 ± 8.89%	44.51 ± 7.58%	77.15 ± 5.13%	22.85 ± 5.13%
Cx, auditory	33	3.89 ± 1.12%	17.59 ± 3.36%	55.07 ± 6.12%	27.33 ± 4.20%	60.87 ± 4.97%	39.12 ± 4.97%
Cx. cingulate	21	4.45 ± 1.77%	17.35 ± 4.02%	49.96 ± 6.57%	32.69 ± 7.01%	64.94 ± 8.53%	35.06 ± 8.53%
Cx. Ectorhinal	12	3.04 ± 0.83%	17.07 ± 4.22%	55.38 ± 12.34%	27.55 ± 9.20%	61.19 ± 6.47%	38.81 ± 6.47%
Cx. entorhinal	25	2.92 ± 1.06%	15.56 ± 3.99%	50.38 ± 7.89%	34.06 ± 5.65%	68.73 ± 5.33%	31.27 ± 5.33%
Cx. frontal	22	3.21 ± 1.05%	21.35 ± 4.51%	48.95 ± 6.67%	29.70 ± 4.74%	58.24 ± 6.23%	41.76 ± 6.23%
Cx, infralimbic	5	2.67 ± 1.58%	15.13 ± 6.18%	47.42 ± 10.20%	37.44 ± 10.12%	71.02 ± 10.30%	28.98 ± 10.30%
Cx, insular	75	3.63 ± 1.98%	15.98 ± 3.49%	55.47 ± 4.79%	28.55 ± 4.46%	64.10 ± 6.93%	35.90 ± 6.93%
Cx, motor	70	3.75 ± 1.26%	19.02 ± 2.96%	48.95 ± 6.34%	32.03 ± 5.59%	62.48 ± 5.52%	37.52 ± 5.52%
Cx. orbital	27	4.09 ± 1.06%	18.87 ± 4.47%	50.86 ± 7.84%	30.27 ± 8.67%	61.12 ± 8.80%	38.88 ± 8.80%
Cx, parietal	23	3.78 ± 1.06%	16.21 ± 1.98%	58.50 ± 4.70%	25.29 ± 3.74%	60.79 ± 3.95%	39.21 ± 3.95%
Cx. peduncular	2	3.98 ± 1.25%	18.83 ± 11.28%	50.98 ± 13.69%	30.19 ± 2.40%	63.37 ± 12.79%	36.63 ± 12.79%
Cx, perirhinal	4	3.44 ± 0.65%	16.45 ± 7.52%	52.97 ± 22.03%	30.59 ± 14.81%	64.69 ± 4.62%	35.31 ± 4.62%
Cx, prelimbic	10	4.09 ± 0.43%	16.72 ± 2.66%	53.75 ± 4.82%	29.53 ± 3.97%	63.80 ± 4.80%	36.20 ± 4.80%
Cx, retrosplenial	21	4.49 ± 1.24%	15.07 ± 3.04%	55.80 ± 7.89%	29.13 ± 7.11%	65.40 ± 7.23%	34.60 ± 7.23%
Cx, somatosensory	39	4.39 ± 1.32%	18.33 ± 3.20%	49.34 ± 4.03%	32.33 ± 4.43%	63.73 ± 6.17%	36.27 ± 6.17%
Cx, temporal	11	3.38 ± 0.70%	16.32 ± 2.58%	62.02 ± 3.19%	21.65 ± 2.74%	57.04 ± 5.61%	42.96 ± 5.61%
Cx, visual	51	4.37 ± 1.28%	16.10 ± 2.78%	59.77 ± 3.97%	24.13 ± 3.31%	60.01 ± 5.48%	39.99 ± 5.48%
Hp, DG, gran	62	2.98 ± 2.33%	3.45 ± 3.44%	89.06 ± 8.26%	7.49 ± 6.17%	66.06 ± 26.77%	33.94 ± 26.77%
Hp, DG, mol	55	3.23 ± 1.96%	27.26 ± 13.77%	13.06 ± 13.50%	59.68 ± 12.38%	69.46 ± 13.48%	30.54 ± 13.48%
Hp, CA1	11	1.99 ± 0.78%	33.35 ± 14.69%	8.64 ± 6.33%	58.19 ± 13.30%	63.85 ± 14.56%	36.15 ± 14.56%
Hp, CA2	2	2.04 ± 0.19%	19.62 ± 5.99%	14.88 ± 2.88%	65.50 ± 3.10%	77.06 ± 6.26%	22.94 ± 6.26%
Hp, CA3	5	2.51 ± 0.64%	18.06 ± 7.39%	21.21 ± 20.39%	60.73 ± 15.39%	77.68 ± 6.43%	22.32 ± 6.43%
Hp, parasubiculum	1	4.29%	19.07%	41.69%	39.24%	67.29%	32.71%
Claustrum	3	2.65 ± 1.05%	9.50 ± 1.81%	14.18 ± 10.75%	76.32 ± 11.32%	88.76 ± 3.01%	11.24 ± 3.01%
Accumbens, core	9	2.22 ± 0.94%	10.34 ± 2.94%	50.61 ± 18.24%	39.05 ± 17.59%	77.45 ± 6.82%	22.55 ± 6.82%
Accumbens, shell	13	2.19 ± 1.38%	11.26 ± 3.81%	51.03 ± 16.25%	37.71 ± 14.18%	76.11 ± 6.88%	23.88 ± 6.88%
Amygdala	30	2.78 ± 1.33%	12.59 ± 5.09%	59.48 ± 9.37%	27.93 ± 8.56%	68.63 ± 11.35%	31.37 ± 11.35%
Pallidum	9	2.10 ± 0.66%	12.54 ± 5.17%	8.39 ± 3.98%	79.07 ± 6.96%	86.25 ± 6.00%	13.75 ± 6.00%
Striatum	70	3.09 ± 0.98%	15.40 ± 3.12%	50.54 ± 7.97%	34.06 ± 7.18%	68.56 ± 5.98%	31.44 ± 5.98%
Thalamus	48	3.31 ± 1.27%	17.08 ± 4.60%	33.03 ± 7.75%	49.89 ± 7.92%	74.38 ± 6.74%	25.62 ± 6.74%
Habenula	5	4.51 ± 2.54%	11.34 ± 4.03%	39.41 ± 10.99%	49.25 ± 12.04%	80.82 ± 7.26%	19.18 ± 7.26%
PAG	8	2.13 ± 1.02%	11.39 ± 3.13%	41.28 ± 8.37%	47.34 ± 8.74%	80.31 ± 5.79%	13.69 ± 5.79%
Inferior coll	47	4.00 ± 1.39%	15.72 ± 5.07%	44.85 ± 12.71%	39.42 ± 9.66%	71.62 ± 6.01%	28.38 ± 6.01%
Superior coll	38	4.21 ± 1.47%	13.56 ± 4.41%	53.73 ± 8.72%	32.71 ± 6.87%	70.86 ± 7.13%	29.14 ± 7.13%
Subst nigra	19	3.92 ± 1.55%	19.67 ± 6.48%	24.44 ± 12.37%	55.89 ± 11.53%	73.74 ± 8.10%	26.26 ± 8.10%
Cb, granular cx	208	3.59 ± 1.93%	1.54 ± 1.12%	95.95 ± 2.03%	2.51 ± 1.72%	60.25 ± 26.64%	39.75 ± 26.64%
Cb, molecular cx	215	2.86 ± 1.54%	17.30 ± 10.64%	32.94 ± 30.74%	49.76 ± 30.43%	65.31 ± 29.61%	34.69 ± 29.61%
Cb, dentate nu	18	5.80 ± 1.33%	23.18 ± 4.31%	19.53 ± 10.75%	57.29 ± 10.49%	70.83 ± 6.21%	29.17 ± 6.21%
Cb nu, other	48	5.10 ± 1.72%	22.45 ± 5.67%	18.70 ± 8.27%	58.85 ± 7.71%	72.39 ± 6.35%	27.61 ± 6.35%
Cochlear nu	20	4.53 ± 2.08%	15.33 ± 4.71%	41.21 ± 12.58%	43.47 ± 10.97%	73.30 ± 7.30%	26.40 ± 7.30%
Gigantocellular nu	19	3.33 ± 0.86%	19.77 ± 3.42%	20.46 ± 7.15%	59.77 ± 8.68%	74.82 ± 5.52%	25.18 ± 5.52%
Inferior olive	5	5.73 ± 1.66%	24.66 ± 3.69%	26.39 ± 15.21%	48.95 ± 15.61%	65.18 ± 9.08%	34.82 ± 9.08%
Pontine nu	21	4.31 ± 1.73%	18.72 ± 5.12%	34.35 ± 6.13%	46.93 ± 6.06%	71.56 ± 7.14%	28.44 ± 7.14%
Reticular nu.	11	3.67 ± 1.24%	20.86 ± 3.65%	21.26 ± 6.32%	57.87 ± 6.32%	73.44 ± 4.39%	26.56 ± 4.39%
Spinal trigeminal nu	5	6.09 ± 2.17%	19.10 ± 4.03%	47.25 ± 12.08%	36.65 ± 10.53%	62.88 ± 9.54%	37.12 ± 9.54%
Superior olive	6	6.26 ± 1.53%	24.42 ± 4.24%	16.55 ± 5.31%	59.03 ± 3.53%	70.85 ± 4.04%	29.15 ± 4.04%
Vestibular nu	9	4.77 ± 1.74%	21.85 ± 7.62%	21.32 ± 13.31%	56.82 ± 12.37%	71.99 ± 9.03%	28.01 ± 9.03%
WM, Cx	90	1.76 ± 1.10%	7.48 ± 4.34%	1.45 ± 2.59%	91.06 ± 4.94%	92.41 ± 4.40%	7.59 ± 4.40%
WM, callosum	31	1.27 ± 0.71%	5.41 ± 2.97%	0.26 ± 0.43%	94.32 ± 2.91%	94.57 ± 2.96%	5.43 ± 2.96%
WM, internal capsule	26	1.76 ± 0.45%	10.17 ± 3.25%	0.44 ± 0.88%	89.38 ± 3.35%	89.78 ± 3.27%	10.22 ± 3.27%
WM, Cb cx	81	2.49 ± 2.32%	12.37 ± 10.88%	6.04 ± 11.78%	81.59 ± 13.95%	87.11 ± 11.30%	12.89 ± 11.30%
WM, Cb peduncle	3	3.02 ± 1.51%	15.01 ± 2.76%	28.30 ± 16.91%	56.69 ± 14.26%	78.88 ± 1.70%	21.12 ± 1.70%

*Values correspond to the mean ± SD across n ROIs in each structure of three rats.*

### Experimental Animals

We obtained three brains of adult male Sprague-Dawley rats from BrainBits (Springfield, IL, United States). According to the supplier, animals were aged 2–2.5 months, weighed 300-400 g, and were housed in a thermally controlled environment at 27°C with *ad libitum* access to chow and water. We opted to only examine male animals for consistency given resource limitations that precluded inclusion of sufficient numbers of both males and females for relevant comparison in this study. The animals were perfused through the heart with 0.9% saline, followed by 4% paraformaldehyde in phosphate buffer, and shipped in 4% paraformaldehyde. Brains were washed in 0.1M phosphate buffer then immersed for 2 days in 30% sucrose in 0.1M phosphate buffer for cryoprotection. Using a sliding microtome (American Optical Corp., Southbridge, MA, United States, Model 860), the ensemble of cerebellum and brainstem were cut into a series of 50 μm thick sagittal sections, and the remaining cerebrum was cut into a series of 50 μm coronal sections. All sections were stored at –20°C in antifreeze solution (30% polyethylene glycol and 30% glycerol in 0.1 M phosphate buffer) until use.

### Immunofluorescence

**FIGURE 1:**
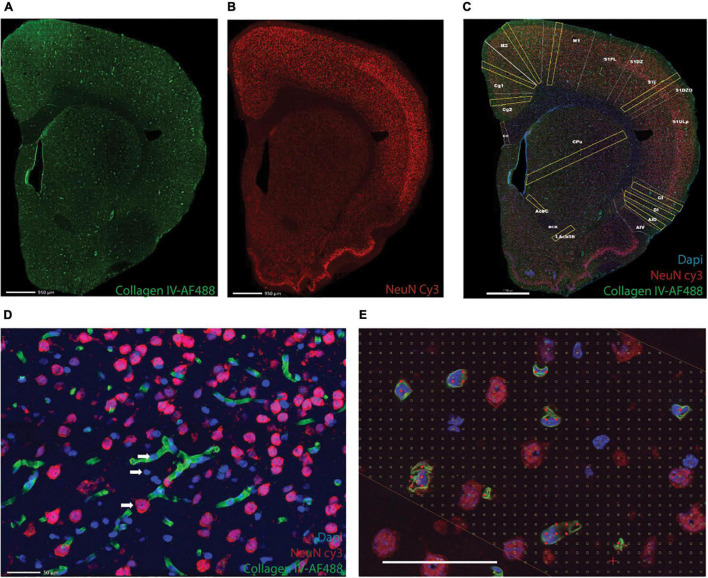
Triple labeling reveals microvasculature, neurons, and all cell nuclei. Shown is an example of a full image montage acquired at 5x magnification of a coronal section of the rat cerebrum labeled with AF 488-anti-collagen IV to reveal microvasculature **(A)**, Cy3-anti-NeuN to reveal neuronal nuclei **(B)**, and counterstained with DAPI to reveal all cell nuclei (**C**, merged images). Scale bars in **(A–C)**, 950 μm. **(D)** Example of an area inside one of the ROIs in **(C)**, imaged using a 63x objective to reveal neurons (red), microvasculature and associated cells (endothelial cell nuclei, in blue, juxtaposed to capillaries, in green), and glial cells (blue). **(E)** Example of a Cavalieri grid used to estimate the microvascular fraction in each ROI. Scale bars in **(D,E)** 50 μm.

One in every eight sections of the cerebrum and one in four of the cerebellum and brainstem were subjected to immunohistochemistry with anti-NeuN antibody to reveal neurons, anti-collagen IV to reveal cells intimately associated with capillaries (vascular fraction), and counterstaining with DAPI (Figures 1A–C). Nuclei that overlapped with capillaries were considered “endothelial cells”; by elimination, cells without anti-NeuN and anti-Collagen IV labeling were counted as glial cells (Figure 1D).

Each section was washed in phosphate buffer (PB), heated for 1 h at 70°C in a 0.1 M solution of boric acid, pH 9.0, then incubated in blocking buffer [phosphate buffer containing 3% normal goat serum (Sigma-Aldrich, Saint Louis, MO, United States, LG9023) and 2% bovine serum albumin (Sigma-Aldrich- A2058)] for 1 h. Sections were then incubated during 48 h at 4°C in blocking buffer containing rabbit polyclonal anti-collagen IV antibody (Abcam, Boston, MA, United States, Ab6586) at a 1:500 dilution, followed by 2 h at 4°C in a 1:500 dilution of Alexa Fluor 488 goat anti-rabbit secondary antibody (Abcam, Ab150077). Sections were only then stained with Cy3-conjugated rabbit anti-NeuN primary polyclonal antibody (Millipore, Burlington, MA, United States, ABN78C3) diluted 1:500 in PB, during 72 h at 4°C under continuous stirring. All sections were labeled with DAPI (4’,6-Diamidino-2-Phenylindole Dilactate, from stock solution at 20 mg/l) to provide counterstaining for identification of brain structures and allow visualization of all cell nuclei in each section. The sections were then mounted on glass slides and coated with Vectashield (Vector Labs, Burlingame, CA, United States, Cat. number H-1400) for viewing under fluorescence microscopy.

### Microscopy

Two-dimensional montages of the entirety of each processed brain section were acquired under magnification with a 5x objective for documentation and careful matching to an atlas (Paxinos and Watson, 2006) for the placement of regions of interest (ROIs). These ROIs, delineated as detailed below, were then imaged at 63x magnification (Plan-ApoChromat objective), which gives a depth of field of 0.5 μm with oil and an optical plane of 0.5 μm, using two Zeiss AxioImager M2 microscopes equipped with Apotome 2 structured illumination (Carl Zeiss, Jena, Germany) operated by StereoInvestigator software (Williston, VT, United States). A total of 135 sections were imaged for the cerebrum and cerebellum/brainstem of three rat brains, respectively: 31 and 23 sections in rat 01, 20 and 17 sections in rat 02, and 31 and 13 sections in rat 03.

### Structures Analyzed

In each of the three rats, we analyzed a total of 53 brain structures in the cerebrum, midbrain, hindbrain and cerebellum (listed in Table 1). In the composite micrograph of each section, we drew a rectangle as long as necessary to span the entirety of each recognizable target structure, taking care to avoid any vessels obviously larger than capillaries. Rectangles placed in cortical structures were at least 0.5 mm wide at the pial surface. Each of these rectangles constituted one ROI. A total of 1734 ROIs were imaged initially across the three animals (respectively, 1,167, 236, and 330), then an additional 392 ROIs were placed in the three animals (respectively, 131, 131, and 130) in 22 structures matching those with reported rates of blood flow and glucose use at rest reported in the literature (Sokoloff et al., 1977; McCulloch et al., 1982; Nakai et al., 1983; Gjedde and Diemer, 1985; Levant and Pazdernik, 2004).

Estimates of local cell densities were obtained by classifying and counting every DAPI-labeled cell contained in each ROI exclusively as neuronal (if it expressed NeuN), “endothelial” (if it did not express NeuN and, was immediately associated with collagen IV-labeled capillaries), or glial (if it neither expressed NeuN nor was immediately associated with capillaries). Numbers of cells were then divided by the area sampled.

Estimates of capillary fraction in the tissue were performed using a Cavalieri probe (Gundersen and Jensen, 1987) using StereoInvestigator software (Microbrightfield, Williston, VT). As established previously (see text footnote 1), a Cavalieri estimator grid with 4 microns distance between points was placed in each ROI for estimating the fractional area occupied by capillaries. Capillary fractional area (which we transformed into percentages) was calculated as the ratio between grid points in the ROI that fell on capillaries identified by anti-collagen IV staining (Figure 1E) and all points in the ROI.

### Statistical Analysis

All analyses were performed in JMP14 PRO, using non-parametric Spearman correlation coefficients to test the correlation between variables, and least-squares regression of raw or log-transformed values to linear functions to determine the slope of power function relationships across variables.

## Results

**FIGURE 2:**
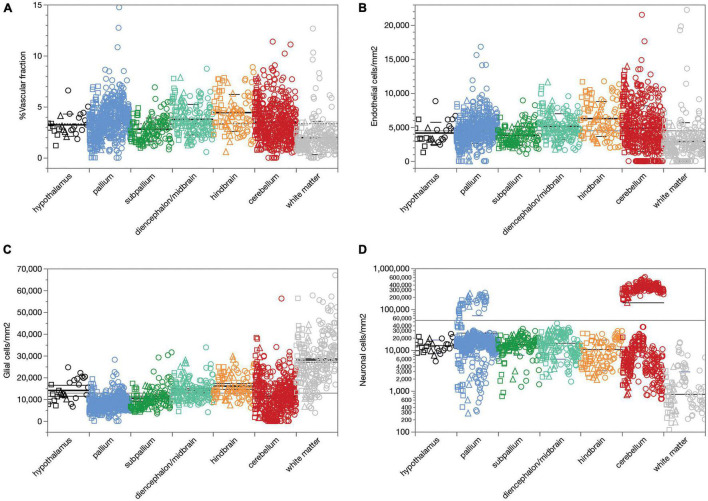
Average capillary fraction, in% of parenchyma surface or volume, and cellular densities across sites and structures in the different brain subdivisions of three rat brains. Different symbols indicate each of three animals (circles, squares, triangles). Bars indicate means and standard deviation for each brain subdivision; long line indicates grand mean across all sites. **(A)** Capillary fraction. Grand mean of 3.36 ± 1.78% (*n* = 1733 sites) indicated by dotted line. Means ± SD: hypothalamus, 3.22 ± 1.09% (*n* = 27 sites); pallium, 3.65 ± 1.63% (*n* = 587 sites); subpallium, 2.80 ± 1.13% (*n* = 133 sites); diencephalon/midbrain, 3.76 ± 1.49% (*n* = 165 sites); hindbrain, 4.42 ± 1.80% (*n* = 96 sites); cerebellum, 3.50 ± 1.89% (*n* = 494 sites); white matter, 1.96 ± 1.62% (*n* = 231 sites). **(B)** Endothelial cell densities. Means ± SD, cells/mm^2^: hypothalamus, 4,122 ± 1,633 (*n* = 27 sites); pallium, 4,609 ± 1,983 (*n* = 587 sites); subpallium, 3,797 ± 1,382 (*n* = 134 sites); diencephalon/midbrain, 5,100 ± 1,911 (*n* = 165 sites); hindbrain, 6,245 ± 2,572 (*n* = 96 sites); cerebellum, 4,817 ± 3,355 (*n* = 494 sites); white matter, 2,877 ± 2,820 (*n* = 231 sites). **(C)** Glial cell densities. Means ± SD, cells/mm^2^: hypothalamus, 14,602 ± 5,027 (*n* = 27 sites); pallium, 8,276 ± 3,171 (*n* = 587 sites); subpallium, 10,436 ± 4,961 (*n* = 134 sites); diencephalon/midbrain, 14,157 ± 4,649 (*n* = 165 sites); hindbrain, 16,024 ± 5,353 (*n* = 96 sites); cerebellum, 11,152 ± 8,010 (*n* = 494 sites); white matter, 28,047 ± 11,309 (*n* = 231 sites). **(D)** Neuronal cell densities. Means ± SD, cells/mm^2^: hypothalamus, 13,140 ± 4,753 (*n* = 27 sites); pallium, 26,463 ± 44,103 (*n* = 587 sites); subpallium, 14,135 ± 6,525 (*n* = 134 sites); diencephalon/midbrain, 14,950 ± 8,866 (*n* = 165 sites); hindbrain, 10,511 ± 7,343 (*n* = 96 sites); cerebellum, 146,162 ± 170,169 (*n* = 494 sites); white matter, 840 ± 2,130 (*n* = 231 sites).

We first analyzed a total of 387,809 cells in 2,301 ROIs located in 53 structures in the brain of three rats. We find that across all 1,734 ROIs, microvasculature amounts to 3.36 ± 1.78% (mean ± standard deviation) of the area of the parenchyma in brain sections, and therefore presumably a similar 3.36 ± 1.78% of the volume of the parenchyma as well (Figure 2A). The standard deviation indicates a coefficient of variation of 53.0% around the mean capillary fraction across all sites, with 50% of sites with fractional microvasculature values between 2.06 and 4.37%. As we found in the mouse brain (Ventura-Antunes and Herculano-Houzel, in review), there is only a ca. fourfold variation in average local capillary fraction measured across structures (Table 1), from the lowest values in the white matter of the corpus callosum (1.27 ± 0.71%) and internal capsule (1.76 ± 0.45%) to the highest values in the superior olive (6.26 ± 1.53%), dentate nucleus of the cerebellum (5.80 ± 1.33%) and superior colliculus (4.21 ± 1.47%).

We find that endothelial cells comprise 14.10 ± 9.11% of all cells and 30.36 ± 18.67% of the non-neuronal cells in the rat brain parenchyma, ranging between 10.88 ± 0.73% of all cells in the nucleus accumbens to 27.26 ± 1.85% in the molecular layer of the hippocampal dentate gyrus (Table 1). As in the mouse brain (Ventura-Antunes and Herculano-Houzel, in review), the percentage of cells in the rat parenchyma that compose the capillary bed is about threefold higher than the microvascular tissue fraction, which suggests that individual endothelial cells are on average smaller than neurons and non-neuronal cells in the tissue.

Similarly, we find that compared to neuronal densities, endothelial cell densities are very stable across structures in the rat brain, at an overall density of 4,504 ± 2,666 cells/mm^2^ across all 1,734 sites analyzed (Figure 2B). The standard deviation indicates a coefficient of variation (CV) of 59.0%, similar to the 53% found for mean capillary fraction, with 50% of the sites with endothelial cell densities between 2,834 and 5,737 cells/mm^2^. As found for capillary fraction, there is a relatively small, ca. 4.5-fold variation in local average endothelial cell densities measured across structures, from the lowest values in the corpus callosum (2,058 ± 1,311 cells/mm^2^) to the highest in the cerebellar dentate nucleus (9,620 ± 2,262 cells/mm^2^; Table 2). Glial cell densities are similarly consistent across structures, with an overall density of 12,975 ± 9,186 cells/mm^2^ across all 1,734 sites, 50% of sites with glial cell densities between 7,095 and 16,207, and a CV of 70.9%, with noticeably higher glial cell densities only in white matter structures (Figure 2C). In contrast, neuronal cell densities are enormously diverse, even when analysis is restricted to gray matter sites (although there are consistently detectable neurons in white matter structures). Overall, we find an average neuronal density of 54,012 ± 111,192 neurons/mm^2^, with a large CV of 205.9%, 50% of sites with neuronal densities between 3,780 and 20,246 neurons/mm^2^, and local averages ranging from as few as 794 ± 772 neurons/mm^2^ in the hippocampal CA1 to as many as 137,624 ± 65,597 and 337,953 ± 71,200 neurons/mm^2^ in the granular layers of the hippocampal dentate gyrus and cerebellar cortex (Table 2 and Figure 2D; notice the logarithmic scale, in comparison to the linear scale in Figures 2A–C).

**TABLE 2:** Cell densities in different structures of the rat brain.

Structure	*n*	Endothelial cells/mm^2^	Neurons/mm^2^	Glial cells/mm^2^	E/N	G/N	G/E
Hypothalamus	27	4,122 ± 1,633	13,140 ± 4,753	14,062 ± 5,027	0.355 ± 0.278	1.193 ± 0.774	3.584 ± 1.011
Cx, auditory	33	4,511 ± 1,350	13,859 ± 2,379	7,019 ± 1,957	0.329 ± 0.097	0.510 ± 0.134	1.594 ± 0.318
Cx. cingulate	21	5,081 ± 1,566	14,732 ± 3,862	9,718 ± 3,234	0.355 ± 0.101	0.679 ± 0.214	2.043 ± 0.883
Cx. ectorhinal	12	4,440 ± 1,467	14,608 ± 4,628	6,974 ± 1,941	0.362 ± 0.280	0.607 ± 0.563	1.643 ± 0.443
Cx. entorhinal	25	3,890 ± 1,175	13,306 ± 4,622	8,649 ± 2,338	0.343 ± 0.253	0.750 ± 0.448	2.293 ± 0.592
Cx. frontal	22	4,890 ± 1,272	11,281 ± 2,608	6,798 ± 1,502	0.455 ± 0.167	0.629 ± 0.185	1.456 ± 0.439
Cx, infralimbic	5	3,445 ± 1,052	11,435 ± 4,532	9,010 ± 3,603	0.337 ± 0.166	0.854 ± 0.417	2.750 ± 1.089
Cx, insular	75	4,170 ± 1,501	14,446 ± 4,026	7,484 ± 2,468	0.293 ± 0.080	0.524 ± 0.124	1.903 ± 0.647
Cx, motor	70	4,816 ± 1,207	12,480 ± 3,333	8,205 ± 2,426	0.399 ± 0.104	0.679 ± 0.204	1.724 ± 0.419
Cx. orbital	27	5,244 ± 1,223	14,509 ± 4,060	8,390 ± 2,031	0.381 ± 0.104	0.659 ± 0.497	1.768 ± 1.007
Cx, parietal	23	5,119 ± 1,229	18,337 ± 3,301	8,008 ± 2,023	0.280 ± 0.052	0.439 ± 0.095	1.576 ± 0.266
Cx. peduncular	2	3,919 ± 347	13,473 ± 9,982	7,297 ± 3,317	0.414 ± 0.332	0.621 ± 0.214	1.907 ± 1.015
Cx, perirhinal	4	4,594 ± 1,299	16,154 ± 7,746	8,556 ± 2,816	0.480 ± 0.553	0.900 ± 1.047	1.868 ± 0.360
Cx, prelimbic	10	4,230 ± 406	13,861 ± 2,530	7,603 ± 1,543	0.316 ± 0.074	0.559 ± 0.123	1.806 ± 0.372
Cx, retrosplenial	21	5,778 ± 1,870	21,002 ± 4,337	11,141 ± 4,014	0.279 ± 0.081	0.550 ± 0.215	2.003 ± 0.582
Cx, somatosensory	39	5,759 ± 1,575	15,333 ± 3,116	10,134 ± 2,625	0.375 ± 0.076	0.664 ± 0.130	1.834 ± 0.479
Cx, temporal	11	4,133 ± 1,224	15,431 ± 2,467	5,403 ± 1,197	0.265 ± 0.051	0.351 ± 0.058	1.365 ± 0.315
Cx, visual	51	5,492 ± 1,636	20,302 ± 4,074	8,138 ± 1,597	0.272 ± 0.060	0.409 ± 0.086	1.544 ± 0.327
Hp, DG, gran	62	4,235 ± 3,835	137,624 ± 65,597	8,512 ± 5,277	0.042 ± 0.048	0.092 ± 0.088	2.335 ± 2.452
Hp, DG, mol	55	3,747 ± 2,010	2,439 ± 3,670	8,913 ± 3,763	2.580 ± 3.229	5.493 ± 5.020	3.217 ± 3.216
Hp, CA1	11	2,709 ± 1,436	794 ± 772	4,697 ± 2,103	3.112 ± 1.855	5.648 ± 2.770	2.138 ± 1.121
Hp, CA2	2	2,824 ± 228	2,246 ± 844	9,750 ± 2,295	1.383 ± 0.671	4.464 ± 0.657	3.527 ± 1.235
Hp, CA3	5	2,914 ± 1,429	3,006 ± 2,712	10,104 ± 5,025	1.135 ± 0.934	3.188 ± 2.154	3.811 ± 1.473
Hp, parasubiculum	1	6,408	14,007	13,183	0.458	0.941	2.057
Claustrum	3	2,883 ± 991	3,939 ± 2,955	22,993 ± 7,424	1.196 ± 1.218	10.105 ± 10.277	8.316 ± 2.386
Accumbens, core	9	3,032 ± 1,091	15,066 ± 6,379	11,494 ± 5,165	0.263 ± 0.221	1.176 ± 1.292	4.074 ± 2.582
Accumbens, shell	13	3,565 ± 1,911	16,195 ± 7,081	12,294 ± 7,085	0.283 ± 0.254	0.991 ± 0.886	3.561 ± 1.478
Amygdala	30	3,724 ± 1,786	18,371 ± 6,437	8,314 ± 2,847	0.224 ± 0.117	0.506 ± 0.263	2.744 ± 1.732
Pallidum	9	2,768 ± 1,010	1,858 ± 812	18,129 ± 5,166	1.819 ± 0.986	11.863 ± 5.976	7.712 ± 4.503
Striatum	70	4,142 ± 1,018	13,832 ± 4,231	9,337 ± 2,994	0.318 ± 0.105	0.720 ± 0.318	2.301 ± 0.659
Thalamus	48	4,421 ± 1,415	8,901 ± 3,655	13,410 ± 4,872	0.553 ± 0.213	1.650 ± 0.678	3.224 ± 1.329
Habenula	5	4,943 ± 1,707	18,709 ± 9,103	21,304 ± 5,562	0.310 ± 0.136	1.476 ± 1.042	4.827 ± 2.100
PAG	8	2,857 ± 947	10,739 ± 3,207	12,215 ± 4,043	0.291 ± 0.127	1.243 ± 0.549	4.626 ± 2.178
Inferior coll	47	5,581 ± 1,635	17,378 ± 8,450	14,105 ± 3,209	0.405 ± 0.222	1.022 ± 0.558	2.702 ± 0.907
Superior coll	38	5,925 ± 2,115	23,712 ± 6,282	14,438 ± 4,433	0.269 ± 0.128	0.652 ± 0.308	2.706 ± 1.187
Subst nigra	19	4,962 ± 2,342	7,529 ± 7,069	14,551 ± 6,111	1.049 ± 0.673	3.005 ± 1.644	3.650 ± 3.596
Cb, granular cx	208	5,174 ± 3,772	337,953 ± 71,200	8,651 ± 6,674	0.016 ± 0.012	0.027 ± 0.019	1.991 ± 2.168
Cb, molecular cx	215	3,627 ± 2,353	6,750 ± 6,857	10,970 ± 7,836	1.218 ± 1.647	4.289 ± 5.388	3.453 ± 3.232
Cb, dentate nu	18	9,620 ± 2,262	7,888 ± 3,778	24,458 ± 8,166	1.517 ± 0.838	3.913 ± 2.274	2.567 ± 0.710
Cb nu, other	48	6,926 ± 2,680	5,893 ± 4,000	17,830 ± 5,037	1.421 ± 0.668	3.686 ± 1.424	2.877 ± 1.219
Cochlear nu	20	6,550 ± 2,565	17,556 ± 6,156	18,669 ± 7,205	0.423 ± 0.216	1.226 ± 0.637	3.183 ± 1.741
Gigantocellular nu	19	4,072 ± 994	4,177 ± 1,524	12,501 ± 4,060	1.051 ± 0.334	3.343 ± 1.413	3.167 ± 0.972
Inferior olive	5	9,104 ± 2,529	10,839 ± 8,994	17,002 ± 2,830	1.209 ± 0.675	2.686 ± 1.952	2.042 ± 0.827
Pontine nu	21	6,543 ± 2,222	12,449 ± 4,828	15,454 ± 3,920	0.577 ± 0.233	1.444 ± 0.507	2.818 ± 1.382
Reticular nu.	11	4,867 ± 2,069	5,038 ± 2,390	13,427 ± 4,806	1.086 ± 0.440	3.055 ± 1.322	2.873 ± 0.719
Spinal trigeminal nu	5	8,598 ± 2,576	20,811 ± 5,625	14,872 ± 5,133	0.445 ± 0.223	0.813 ± 0.502	1.814 ± 0.591
Superior olive	6	8,874 ± 1,630	6,012 ± 1,972	21,492 ± 2,274	1.715 ± 0.937	4.052 ± 1.939	2.487 ± 0.499
Vestibular nu	9	6,493 ± 2,588	7,489 ± 7,668	16,207 ± 3,407	1.433 ± 0.939	3.731 ± 2.253	3.085 ± 1.977
WM, Cx	90	2,660 ± 1,690	495 ± 910	33,080 ± 9,229	4.224 ± 3.667	59.904 ± 45.703	14.930 ± 8.628
WM, callosum	31	2,058 ± 1,311	103 ± 171	35,160 ± 10,226	5.556 ± 2.920	109.389 ± 24.852	21.272 ± 10.696
WM, internal capsule	26	2,712 ± 945	112 ± 209	24,450 ± 6,806	10.000 ± 6.245	86,518 ± 54.539	10.026 ± 4.452
WM, Cb cx	81	3,425 ± 4,241	1,381 ± 2,494	21,305 ± 10,747	1.319 ± 1.646	9.283 ± 8.496	7.751 ± 8.720
WM, Cb peduncle	3	4,519 ± 1,456	10,519 ± 7,884	16,798 ± 5,204	0.904 ± 0.950	3.533 ± 3.873	3.754 ± 0.366

*Values correspond to the mean ± SD across n ROIs in each structure of three rats.*

**FIGURE 3:**
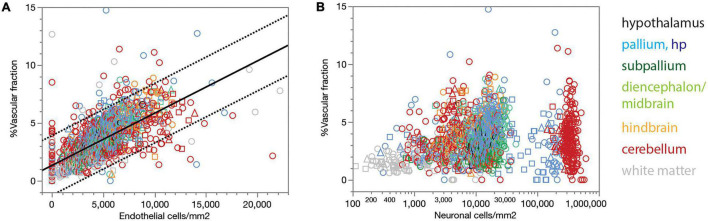
Local vascular fraction increases with endothelial cell density but does not accompany local neuronal density consistently across brain structures. **(A)** Local vascular fraction varies as a linear function of endothelial cell densities across all ROIs of all three animals, and also across sites within each brain subdivision. Spearman correlation across all sites: ρ = 0.742, *p* < 0.0001. Spearman correlation within brain subdivisions: Hypothalamus, ρ = 0.790, *p* < 0.0001; pallium, ρ = 0.711, *p* < 0.0001; subpallium, ρ = 0.765, *p* < 0.0001; diencephalon/midbrain, ρ = 0.801, *p* < 0.0001; hindbrain, ρ = 0.806, *p* < 0.0001; cerebellum, ρ = 0.654, *p* < 0.0001; white matter, ρ = 0.670, *p* < 0.0001. **(B)** Local vascular fraction correlates significantly with local neuronal density across sites within several brain structures, but not consistently across structures, such that highly disparate neuronal densities occur with similar vascular fractions across structures. Spearman correlation across all sites: ρ = 0.286, *p* < 0.0001. Spearman correlation within brain subdivisions: Hypothalamus, ρ = 0.437, *p* = 0.0226; non-hippocampal pallium, ρ = 0.419, *p* < 0.0001; whole pallium, ρ = 0.250, *p* < 0.0001; subpallium, ρ = 0.099, *p* = 0.2594; diencephalon/midbrain, ρ = 0.243, *p* = 0.0017; hindbrain, ρ = 0.248, *p* = 0.0005; cerebellum, ρ = 0.027, *p* = 0.5511; white matter, ρ = –0.002, *p* = 0.9766.

Our estimates of the local capillary area fraction correlate strongly, and linearly, with endothelial cell density across all sites (Spearman correlation, ρ = 0.742, *p* < 0.0001; Figure 3A, linear fit, *r*^2^ = 0.457, *p* < 0.0001). Similarly strong correlations across all three animals examined are found for each brain subdivision (Figure 3A), which validates the use of endothelial cell density as a surrogate indicator of capillary density across all sites within the brain of a species.

Although the microvasculature is the sole source of energy to neurons in the brain, we find that local capillary volume fraction is only poorly correlated with neuronal density across all brain sites (Spearman correlation, ρ = 0.286, *p* < 0.0001), and clearly not in a universal manner (Figure 3B). Besides the much larger variation in neuronal density than in capillary volume fraction, we find that neuronal densities spanning two orders of magnitude across structures occur with similar (and similarly small) capillary fractions (Figure 3B). Importantly, while significant positive correlations exist between local capillary fraction and neuronal density across sites *within* some structures, notably the hypothalamus and non-hippocampal pallium, they are not evident within *all* structures in the rat brain, and also not aligned *across* brain structures (Figure 3B).

**FIGURE 4:**
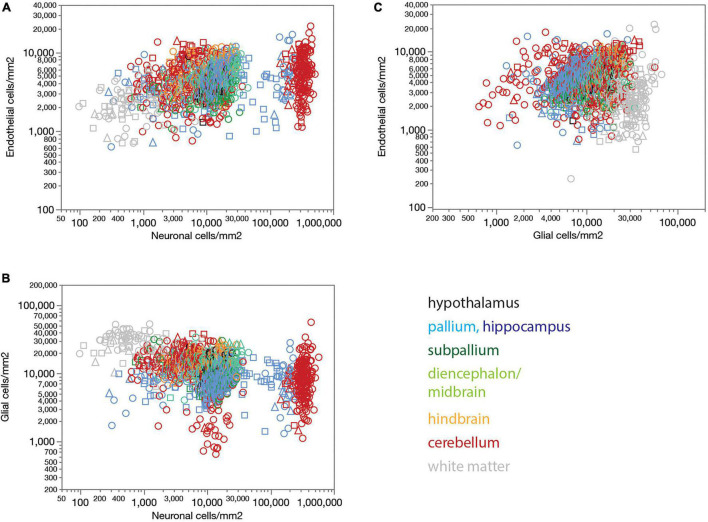
Endothelial, neuronal, and glial cell densities are not universally correlated across structures in the rat brain. **(A)** Local endothelial cell density correlates significantly with local neuronal density across sites within several brain structures, but not consistently across structures, such that highly disparate neuronal densities occur with similar endothelial cell densities across structures. Spearman correlation across all sites: ρ = 0.283, *p* < 0.0001. Spearman correlation within brain subdivisions: Hypothalamus, ρ = 0.434, *p* = 0.0239; pallium, ρ = 0.332, *p* < 0.0001; non-hippocampal pallium, ρ = 0.484, *p* < 0.0001; subpallium, ρ = 0.195, *p* = 0.0242; diencephalon/midbrain, ρ = 0.369, *p* < 0.0001; hindbrain, ρ = 0.469, *p* < 0.0001; cerebellum, ρ = 0.053, *p* < 0.0001; white matter, ρ = –0.097, *p* = 0.1436. **(B)** Local glial cell density correlates significantly with local neuronal density across sites within several brain structures, but not consistently across structures, such that highly disparate neuronal densities occur with similar endothelial cell densities across structures. Spearman correlation across all sites: ρ = –0.420, *p* < 0.0001. Spearman correlation within brain subdivisions: Hypothalamus, ρ = 0.392, *p* = 0.0432; pallium, ρ = 0.265, *p* < 0.0001; non-hippocampal pallium, ρ = 0.441, *p* < 0.0001; subpallium, ρ = –0.098, *p* = 0.2597; diencephalon/midbrain, ρ = 0.280, *p* = 0.0003; hindbrain, ρ = 0.322, *p* = 0.0014; cerebellum, ρ = –0.427, *p* < 0.0001; white matter, ρ = –0.128, *p* = 0.0529. **(C)** Local endothelial cell density correlates significantly with local glial density across sites within several brain structures, notably in the gray matter, but not consistently across structures. Spearman correlation across all sites: ρ = 0.057, *p* = 0.0182. Spearman correlation within brain subdivisions: Hypothalamus, ρ = 0.748, *p* < 0.0001; pallium, ρ = 0.405, *p* < 0.0001; non-hippocampal pallium, ρ = 0.547, *p* < 0.0001; subpallium, ρ = 0.182, *p* = 0.0355; diencephalon/midbrain, ρ = 0.339, *p* < 0.0001; hindbrain, ρ = 0.554, *p* < 0.0001; cerebellum, ρ = 0.117, *p* < 0.0001; white matter, ρ = 0.113, *p* = 0.0854; non-cortical gray matter, ρ = 0.550, *p* < 0.0001.

Local endothelial cell densities, like local capillary volume fractions, are not consistently correlated with local neuronal densities across brain sites (Figure 4A). Although higher local neuronal densities are significantly positively correlated with higher local endothelial cell densities within several structures, notably the hypothalamus, non-hippocampal pallium and hindbrain (see figure legend), the correlation across all sites and structures is only poor. Indeed, the entire range of local neuronal densities occurs across structures in the rat brain that share similar endothelial cell densities (Figure 4A).

Local glial cell densities are also much less variable than neuronal densities across brain structures and species. There is a detectable negative correlation between local glial and neuronal densities across all structures and animals in the whole dataset, but no consistent trend within each structure (Figure 4B). Moreover, correlations within brain structures have the opposite sign, with no shared, universal relationship between neuronal and glial cell densities. As found for local endothelial cell densities, the entire, wide range of local neuronal densities occurs across sites with similar local glial cell densities (Figure 4B).

Interestingly, while there is no detectable universal correlation between local glial and endothelial cell densities across all structures, densities of these two subtypes of non-neuronal cells are strongly correlated within and across gray matter structures, though not white matter structures (Figure 4C).

**FIGURE 5:**
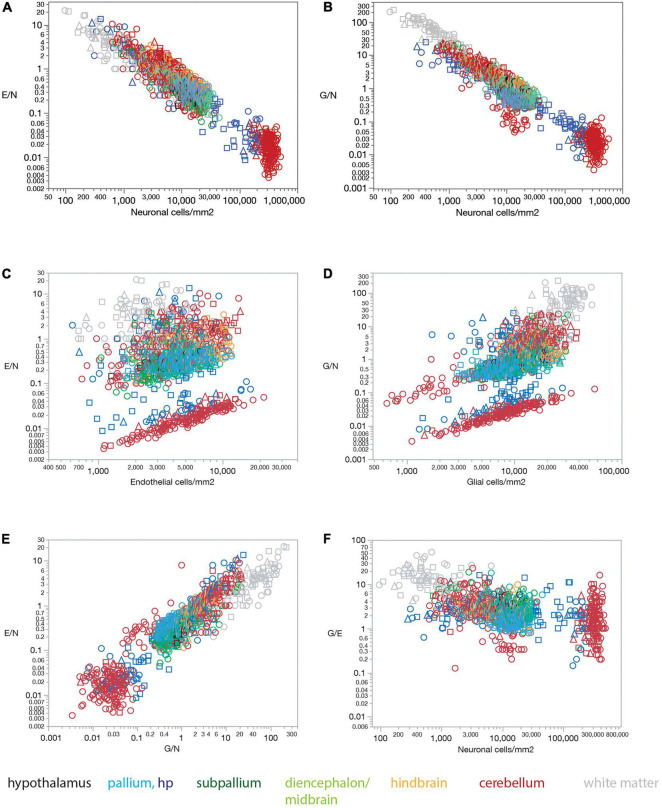
Ratios of endothelial and glial cells per neuron increase with decreasing local neuronal density across sites and structures in all three rat brains. **(A)** The ratio of endothelial cells per neuron E/N varies uniformly with neuronal density across brain sites and structures. Spearman correlation across all sites: ρ = –0.833, *p* < 0.0001. Spearman correlation within brain subdivisions: Hypothalamus, ρ = –0.451, *p* = 0.0182; pallium, ρ = –0.688, *p* < 0.0001; non-hippocampal pallium, ρ = –0.451, *p* < 0.0001; subpallium, ρ = –0.747, *p* < 0.0001; diencephalon/midbrain, ρ = –0.813, p < 0.0001; hindbrain, ρ = –0.847, *p* < 0.0001; cerebellum, ρ = –0.822, *p* < 0.0001; white matter, ρ = –0.867, *p* < 0.0001. **(B)** The ratio of glial cells per neuron G/N varies uniformly with neuronal density across brain sites and structures. Spearman correlation across all sites: ρ = –0.873, *p* < 0.0001. Spearman correlation within brain subdivisions: Hypothalamus, ρ = –0.496, *p* = 0.0085; pallium, ρ = –0.719, *p* < 0.0001; non-hippocampal pallium, ρ = –0.458, *p* < 0.0001; subpallium, ρ = –0.690, *p* < 0.0001; diencephalon/midbrain, ρ = –0.885, *p* < 0.0001; hindbrain, ρ = –0.907, *p* < 0.0001; cerebellum, ρ = –0.829, *p* < 0.0001; white matter, ρ = –0.953, *p* < 0.0001. **(C)** The ratio of endothelial cells per neuron E/N does not vary uniformly with endothelial cell density across brain sites and structures. Spearman correlation across all sites: ρ = 0.202, *p* < 0.0001. Spearman correlation within brain subdivisions: Hypothalamus, ρ = 0.482, *p* = 0.0108; pallium, ρ = 0.310, *p* < 0.0001; non-hippocampal pallium, ρ = 0.507, *p* < 0.0001; subpallium, ρ = 0.419, *p* < 0.0001; diencephalon/midbrain, ρ = 0.192, *p* = 0.0133; hindbrain, ρ = 0.033, *p* = 0.7536; cerebellum, ρ = 0.411, *p* < 0.0001; white matter, ρ = 0.377, *p* = 0.0001. **(D)** The ratio of glial cells per neuron G/N does not vary uniformly with glial cell density across brain sites and structures. Spearman correlation across all sites: ρ = 0.664, *p* < 0.0001. Spearman correlation within brain subdivisions: Hypothalamus, ρ = 0.507, *p* = 0.0069; pallium, ρ = 0.360, *p* < 0.0001; non-hippocampal pallium, ρ = 0.544, *p* < 0.0001; subpallium, ρ = 0.721, *p* < 0.0001; diencephalon/midbrain, ρ = 0.161, *p* = 0.0389; hindbrain, ρ = 0.063, *p* = 0.5401; cerebellum, ρ = 0.752, *p* < 0.0001; white matter, ρ = 0.586, *p* < 0.0001. **(E)** The ratio of endothelial cells per neuron E/N varies uniformly with the ratio of glial cells per neuron G/N across brain sites and structures. Spearman correlation across all sites: ρ = 0.834, *p* < 0.0001. Spearman correlation within brain subdivisions: Hypothalamus, ρ = 0.670, *p* < 0.0001; pallium, ρ = 0.747, *p* < 0.0001; non-hippocampal pallium, ρ = 0.574, *p* < 0.0001; subpallium, ρ = 0.705, *p* < 0.0001; diencephalon/midbrain, ρ = 0.803, *p* < 0.0001; hindbrain, ρ = 0.875, *p* < 0.0001; cerebellum, ρ = 0.736, *p* < 0.0001; white matter, ρ = 0.843, *p* < 0.0001. **(F)** The ratio of glial cells per endothelial cell G/E remains relatively constant across sites and brain subdivisions with different neuronal densities. Spearman correlation across all sites: ρ = –0.515, *p* < 0.0001 (average G/E, 3.854 ± 5.212). Spearman correlation within brain subdivisions: Hypothalamus, ρ = 0.012, *p* = 0.9542 (average G/E, 3.584 ± 1.011); pallium, ρ = –0.121, *p* = 0.0037 (average G/E, 1.994 ± 1.418); non-hippocampal pallium, ρ = –0.019, *p* = 0.6908 (average G/E, 1.780 ± 0.593); subpallium, ρ = –0.170, *p* = 0.0498 (average G/E, 3.139 ± 2.299); diencephalon/midbrain, ρ = –0.108, *p* = 0.1687 (average G/E, 3.122 ± 1.763); hindbrain, ρ = –0.263, *p* = 0.0095 (average G/E, 2.881 ± 1.332); cerebellum, ρ = –0.465, *p* < 0.0001 (average G/E, 2.769 ± 2.683); white matter, ρ = 0.008, *p* = 0.9032 (average G/E, 12.936 ± 9.692).

The lack of a universal correlation between local densities of endothelial cells (or vascular fraction) and neuronal densities across brain structures in the rat, combined with the relatively uniform densities of endothelial cells, results in a universal relationship between the ratio of endothelial cells per neuron (E/N) and local neuronal densities that span over three orders of magnitude across brain structures (Figure 5A). E/N values scale across structures, and even within each structure, accompanying local neuronal density, such that the lower the local density of neurons, the larger the E/N ratio (Figure 5A). Thus, in order of decreasing neuronal density, there are on average over 60 neurons per endothelial cell in the cerebellar granular layer (E/N 0.016 ± 0.012), 24 neurons per endothelial cell in the hippocampal granular layer of the dentate gyrus (E/N 0.042 ± 0.048), 3 neurons per endothelial cell in the hypothalamus and non-hippocampal pallium (E/N 0.355 ± 0.278, 0.339 ± 0.134, respectively), 2.5 neurons per endothelial cell in the subpallium (E/N 0.411 ± 0.511), 2 neurons per endothelial cell in the diencephalon/midbrain (E/N 0.483 ± 0.367), just over 1 neuron per endothelial cell in the hindbrain (E/N 0.875 ± 0.606), 1.218 ± 1.647 endothelial cells for every neuron in the cerebellar molecular layer, and 2.506 ± 2.897 endothelial cells for every neuron in hippocampal sites excluding the granular layer of the dentate gyrus.

Similarly, and as reported previously (Herculano-Houzel, 2014), the lack of a universal correlation between local densities of glial and neuronal cell densities across brain structures in the rat, combined with the relatively uniform densities of glial cells, results in a universal relationship between the ratio of glial cells per neuron (G/N) and local neuronal densities across brain structures (Figure 5B).

In contrast, the ratio of endothelial cells per neuron (E/N) and the ratio of glial cells per neuron (G/N) fail to vary consistently with endothelial cell density (Figure 5C) or glial cell density (Figure 5D). Thus, we find that the lower the local density of neurons, the larger the E/N and G/N ratios (Figures 5A,B), in a manner that applies for grey matter structures, white matter structures, and both together. Such universality strongly supports and extends to the E/N ratio our previous model that proposes that with relatively constant densities of non-neuronal cells, the relevant variable for determining G/N is simply local neuronal density (Herculano-Houzel, 2014; Mota and Herculano-Houzel, 2014). Because local neuronal density correlates with the inverse of average local neuronal cell size (estimated for entire cells, including soma and all arbors; Mota and Herculano-Houzel, 2014), these findings imply that the fewer and larger neurons are at a brain site, the more endothelial and glial cells are available per neuron.

In further support of local neuronal densities as the sole, or main, variable of consequence to determining cell type ratios across brain sites, we find that local E/N and G/N ratios are strongly, directly, and universally correlated across brain sites, such that sites with lower neuronal densities (that is, larger neurons) have both more endothelial and glial cells for every neuron (Figure 5E). As expected from this finding that E/N and G/N increase together, the G/E ratio remains fairly constant across sites and structures as neuronal density varies, at an overall average of typically about 2–3 glial cells per endothelial cell in gray matter structures, except for a much larger ratio of 13 glial cells per endothelial cell in the white matter (Figure 5F).

**FIGURE 6:**
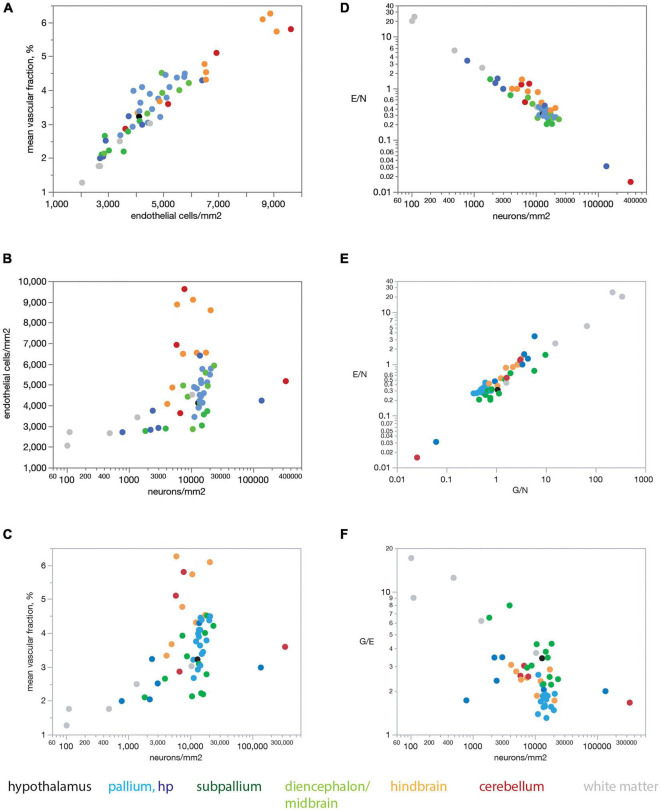
Mean endothelial cell densities correlate with mean capillary fraction but not neuronal density across structures in the rat brain, resulting in E/N ratios that correlate with neuronal density and G/N ratios across structures. Each point represents the average value for one of the brain structures as listed in Tables 1, 2. Data points are color-coded according to the key below the graphs. **(A)** Mean capillary fraction varies as a linear function of endothelial cell density across structures in the rat brain (Spearman correlation ρ = 0.936, *p* < 0.0001; linear fit, *r*^2^ = 0.888, *p* < 0.0001). **(B)** Weak correlation between mean endothelial cell density and mean neuronal density across structures in the rat brain (Spearman correlation ρ = 0.440, *p* = 0.0001). **(C)** Weak correlation between mean capillary fraction and mean neuronal density across structures in the rat brain (Spearman correlation ρ = 0.480, *p* = 0.0010). **(D)** Mean ratio of endothelial cells per neuron (E/N) decreases uniformly with increasing neuronal cell density across brain sites and structures (Spearman correlation ρ = –0.878, *p* < 0.0001; power fit, *r*^2^ = 0.943, *p* < 0.0001). **(E)** The ratio of endothelial cells per neuron E/N varies uniformly and linearly with the ratio of glial cells per neuron G/N across brain structures (Spearman correlation ρ = 0.860, *p* < 0.0001; power fit, *r*^2^ = 0.891, *p* < 0.0001). **(F)** The average ratio of glial cells per endothelial cell G/E is correlated with average neuronal densities across brain structures (Spearman correlation ρ = –0.550, *p* < 0.0001).

Figure 6 shows that our findings also apply when data are averaged across all sites within each brain structure before analysis. Mean capillary fraction is strongly and linearly correlated with endothelial cell density across brain structures (Figure 6A), but there is only a weak correlation between either mean capillary fraction or mean endothelial cell density and mean neuronal density across structures (Figures 6B,C). This analysis also makes evident that while mean capillary fraction and endothelial cell densities are highest in hindbrain and cerebellar structures, and lowest in white matter structures (Figures 6A–C), there is at least a 30-fold variation in average neuronal density across structures whose mean average capillary fraction varies only twofold, between 2 and 4% of the tissue (Figure 6C). As a consequence of the much larger variation in neuronal density than in endothelial (or glial) cell density across brain structures, the E/N ratio decreases tightly together with increasing neuronal densities (Figure 6D), although with clearly larger E/N ratios in hindbrain and cerebellar structures (consistently with the larger vascular fraction of these structures), and E/N increases together with increasing G/N ratios across structures (Figure 6E), resulting in average G/E ratios of between 1.5 and 4 across structures (Figure 6F).

**FIGURE 7:**
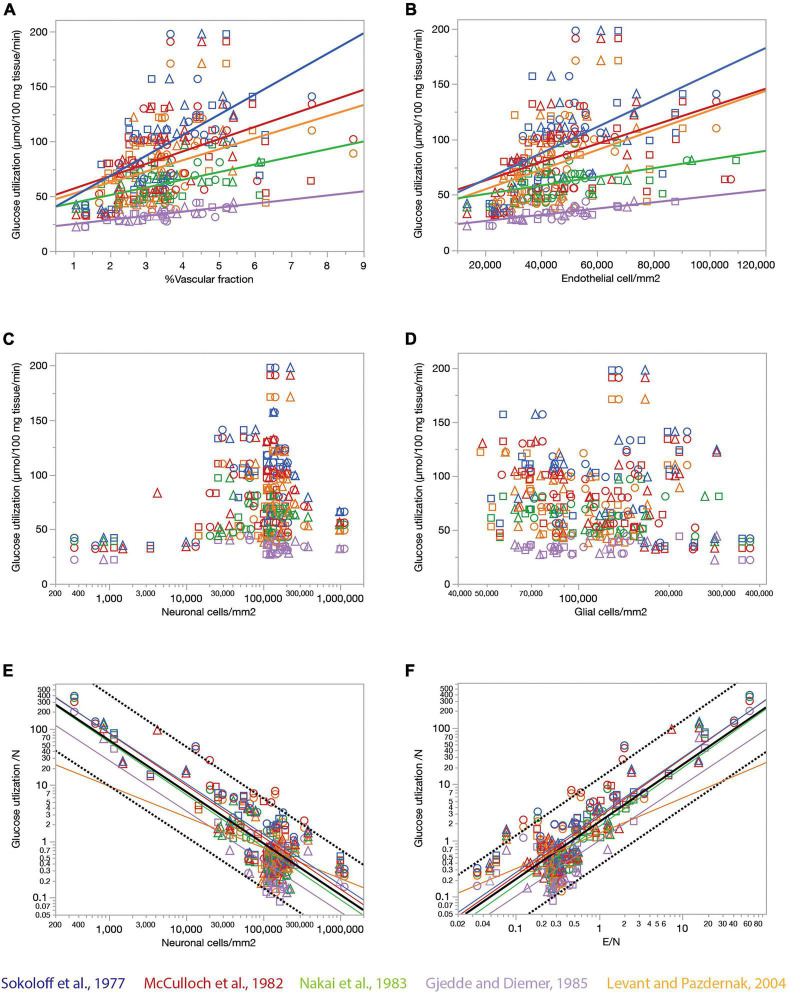
Relationship between local rates of glucose utilization at rest (data from the literature) and capillary fraction, cell densities, and E/N ratio (our data). Each data point refers to one brain structure in the list below. Each dataset is depicted in a different color according to the key. Data from each of three rats are shown separately as indicated by the symbols (circles, triangles, and squares). **(A)** Local rates of glucose utilization reported in the literature correlate with capillary fraction in the same structures in our data. Linear fits are plotted for each dataset. All linear correlations are significant (*p* < 0.0001 unless noted otherwise), with *r*^2^ = 0.450 (Gjedde and Diemer, 1985), 0.381 (Sokoloff et al., 1977), 0.289 (Nakai et al., 1983), 0.211 (McCulloch et al., 1982), and 0.157, *p* = 0.0006 (Levant and Pazdernik, 2004). Spearman correlation coefficients (all values of *p* < 0.0001 unless noted otherwise): ρ = 0.671 (Sokoloff et al., 1977), ρ = 0.610 (Gjedde and Diemer, 1985); ρ = 0.575 (McCulloch et al., 1982); ρ = 0.511 (Nakai et al., 1983); ρ = 0.439, *p* = 0.0001 (Levant and Pazdernik, 2004); ρ = 0.446 (all data). **(B)** Local rates of glucose utilization reported in the literature are linearly correlated with local density of endothelial cells in our data. All linear correlations are significant (*p* < 0.0001 unless noted otherwise), with *r*^2^ = 0.457 (Gjedde and Diemer, 1985), 0.299 (Sokoloff et al., 1977), 0.222 (Nakai et al., 1983), 0.205 (McCulloch et al., 1982) and 0.180, *p* = 0.0002 (Levant and Pazdernik, 2004). Spearman correlation coefficients (all values of *p* < 0.0001): ρ = 0.622 (Sokoloff et al., 1977), ρ = 0.609 (Gjedde and Diemer, 1985); ρ = 0.604 (McCulloch et al., 1982); ρ = 0.510 (Nakai et al., 1983); ρ = 0.480 (Levant and Pazdernik, 2004); ρ = 0.445 (all data). **(C,D)** Local rates of glucose utilization are not significantly correlated with neuronal density (**C**; all values of *p* > = 0.01) or with glial cell density (**D**; all values of *p* > = 0.01) across structures. **(E)** The estimated rate of glucose utilization per neuron decreases linearly with neuronal density. Power fit exponent, *r*^2^ (all *p*-values < 0.0001): –0.896 ± 0.089, *r*^2^ = 0.722 (Gjedde and Diemer, 1985); –0.898 ± 0.065, *r*^2^ = 0.771 (Sokoloff et al., 1977); –0.922 ± 0.066, *r*^2^ = 0.699 (McCulloch et al., 1982); –0.932 ± 0.076, *r*^2^ = 0.729 (Nakai et al., 1983); –0.546 ± 0.133, *r*^2^ = 0.197, *p* = 0.0001 (Levant and Pazdernik, 2004); –0.913 ± 0.037, *r*^2^ = 0.662 (all data). Spearman correlation coefficients (all values of *p* < 0.0001 unless noted otherwise): ρ = –0.651 (Sokoloff et al., 1977), ρ = –0.627, *p* = 0.0370 (Gjedde and Diemer, 1985); ρ = –0.641 (McCulloch et al., 1982); ρ = –0.664 (Nakai et al., 1983); ρ = –0.207, *p* = 0.0837 (Levant and Pazdernik, 2004); ρ = –0.524 (all data). **(F)** The estimated rate of glucose utilization per neuron increases linearly with the ratio of endothelial cells per neuron. Power fit exponent, *r*^2^ (all *p*-values < 0.0001): 0.986 ± 0.105, *r*^2^ = 0.694 (Gjedde and Diemer, 1985); 1.021 ± 0.070, *r*^2^ = 0.791 (Sokoloff et al., 1977); 1.040 ± 0.069, *r*^2^ = 0.730 (McCulloch et al., 1982); 1.041 ± 0.085, *r*^2^ = 0.727 (Nakai et al., 1983); 0.626 ± 0.120, *r*^2^ = 0.284 (Levant and Pazdernik, 2004); 1.016 ± 0.039, *r*^2^ = 0.683 (all data). Spearman correlation coefficients (all values of *p* < 0.0001 unless noted otherwise): ρ = 0.778 (Sokoloff et al., 1977), ρ = 0.446, *p* = 0.0035 (Gjedde and Diemer, 1985); ρ = 0.732 (McCulloch et al., 1982); ρ = 0.749 (Nakai et al., 1983); ρ = 0.345, *p* = 0.0032 (Levant and Pazdernik, 2004); ρ = 0.630 (all data). Structures analyzed: amygdala, auditory cortex, cerebellar cortex, cerebellar dentate nucleus, other cerebellar nuclei, cerebellar white matter, cochlear nucleus, corpus callosum, entorhinal cortex, globus pallidus, habenula (lateral), hippocampus, hypothalamus, inferior colliculus, inferior olive, insular cortex, nucleus accumbens, sensorimotor cortex, superior colliculus, superior olive, striatum, substantia nigra, thalamus, vestibular nucleus, visual cortex.

Endothelial cell densities and capillary volume fraction are easily quantified in fixed brains, and could constitute measurements of great scientific value for examining brain metabolism if they served as proxies of measurements of brain metabolism at rest that can only be obtained in living brains. To address this possibility, we compared data we acquired from 392 ROIs in 22 structures in the brains of three rats selected to match sites with published data of rates of blood flow and glucose use in the non-stimulated (“resting”) rat brain acquired in five independent studies (Sokoloff et al., 1977; McCulloch et al., 1982; Nakai et al., 1983; Gjedde and Diemer, 1985; Levant and Pazdernik, 2004). Figure 7 shows that local rates of resting glucose utilization measured with autoradiographic methods by other groups are strongly and consistently correlated with our measured local capillary fraction (Figure 7A) and endothelial cell densities (Figure 7B), even though the absolute values of local rates of glucose use disagree across studies (different colors in Figure 7). Importantly, we find that local rates of glucose use at rest are not significantly correlated with either neuronal (Figure 7C) or glial cell densities (Figure 7D) measured in the same locations in the rat brain. These data suggest that local endothelial cell densities and capillary fraction are valid proxies for local rates of glucose use in the rat brain at rest, and that these rates bear no relationship with the numbers of neurons or glial cells present in the volume measured.

Our data also allow the estimation of an average rate of glucose utilization per neuronal unit (that is, one neuron and its associated glial cells) and how it varies across sites. Strikingly, we find that the average rate of glucose use per neuronal unit varies uniformly, and negatively, with local average neuronal density across the various sites in the rat brain, such that the smaller the local neuronal density, the higher the rate of glucose use per neuron (Figure 7E). At the same time, the rate of glucose use per neuronal unit increases together, and linearly, with the ratio of endothelial cells per neuron across brain sites (Figure 7F). Taken together, these results suggest that given the fairly even distribution of capillary densities across structures in the rat brain, the more the neurons found in a certain volume, sharing the blood supplied by the capillaries in that volume, the less glucose is used per neuronal unit per unit time in the resting brain.

**FIGURE 8:**
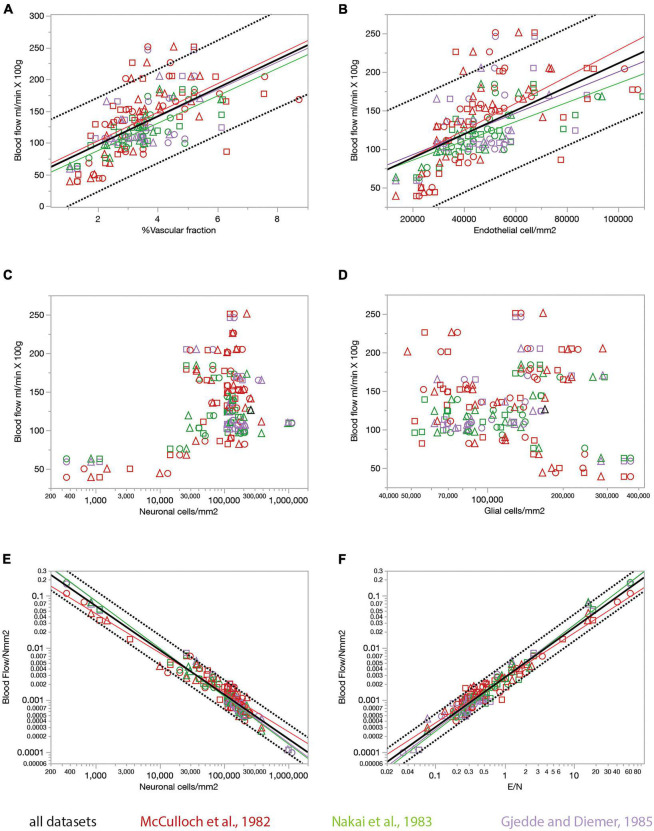
Relationship between local rates of blood flow at rest (data from the literature) and capillary fraction, cell densities, and E/N ratio (our data). Each data point refers to one brain structure in the list below. Each dataset is depicted in a different color according to the key. Data from each of three rats are shown separately as indicated by the symbols (circles, triangles, and squares). **(A)** Local rates of blood flow at rest reported in the literature correlate with capillary fraction in the same structures in our data. Linear fits are plotted for each dataset. All linear correlations are significant (*p* < 0.0001 unless noted otherwise), with *r*^2^ = 0.310, *p* = 0.0002 (Gjedde and Diemer, 1985), 0.620 (Nakai et al., 1983), 0.376 (McCulloch et al., 1982), and 0.392 (all data, plotted). Spearman correlation coefficients (all values of *p* < 0.0001 unless noted otherwise): ρ = 0.546, p = 0.0002 (Gjedde and Diemer, 1985); ρ = 0.740 (McCulloch et al., 1982); ρ = 0.774 (Nakai et al., 1983); ρ = 0.673 (all data). **(B)** Local rates of blood flow at rest reported in the literature are linearly correlated with local density of endothelial cells in our data. All linear correlations are significant (*p* < 0.0001 unless noted otherwise), with *r*^2^ = 0.223, *p* = 0.0018 (Gjedde and Diemer, 1985), 0.524 (Nakai et al., 1983), 0.397 (McCulloch et al., 1982), and 0.364 (all data). Spearman correlation coefficients (all values of *p* < 0.0001): ρ = 0.468, *p* = 0.0022 (Gjedde and Diemer, 1985); ρ = 0.764 (McCulloch et al., 1982); ρ = 0.761 (Nakai et al., 1983); ρ = 0.661 (all data). **(C,D)** Local rates of blood flow at rest are not significantly correlated with neuronal density (**C**, *p* = 0.8381 and 0.4166 for the Gjedde and Nakai datasets, respectively, though ρ = 0.375, *p* = 0.0010 for the McCulloch dataset) or with glial cell density (**D**; all values of *p* > = 0.08) across structures. **(E)** The estimated rate of blood flow at rest per neuron decreases linearly with neuronal density. Power fit exponent, *r*^2^ (all *p*-values < 0.0001): –0.907 ± 0.030, *r*^2^ = 0.960 (Gjedde and Diemer, 1985); –0.756 ± 0.028, *r*^2^ = 0.913 (McCulloch et al., 1982); –0.917 ± 0.023, *r*^2^ = 0.969 (Nakai et al., 1983); –0.546 ± 0.133, –0.852 ± 0.017, *r*^2^ = 0.939 (all data). Spearman correlation coefficients (all values of *p* < 0.0001 unless noted otherwise): ρ = –0.874 (Gjedde and Diemer, 1985); ρ = –0.890 (McCulloch et al., 1982); ρ = –0.964 (Nakai et al., 1983); ρ = –0.908 (all data). **(F)** The estimated rate of blood flow at rest per neuron increases linearly with the ratio of endothelial cells per neuron. Power fit exponent, *r*^2^ (all *p*-values < 0.0001): 1.009 ± 0.036, *r*^2^ = 0.954 (Gjedde and Diemer, 1985); 0.866 ± 0.026, *r*^2^ = 0.941 (McCulloch et al., 1982); 1.023 ± 0.024, *r*^2^ = 0.973 (Nakai et al., 1983); 0.957 ± 0.017, *r*^2^ = 0.951 (all data). Spearman correlation coefficients (all values of *p* < 0.0001 unless noted otherwise): ρ = 0.863 (Gjedde and Diemer, 1985); ρ = 0.913 (McCulloch et al., 1982); ρ = 0.960 (Nakai et al., 1983); ρ = 0.921 (all data). Structures analyzed: amygdala, auditory cortex, cerebellar cortex, cerebellar dentate nucleus, other cerebellar nuclei, cerebellar white matter, cochlear nucleus, corpus callosum, entorhinal cortex, globus pallidus, habenula (lateral), hippocampus, hypothalamus, inferior colliculus, inferior olive, insular cortex, nucleus accumbens, sensorimotor cortex, superior colliculus, superior olive, striatum, substantia nigra, thalamus, vestibular nucleus, visual cortex.

The rate of glucose use by brain structures may or may not be limited by the rate of glucose supply by blood flow to the brain, for instance because of the possibility of variation in expression of glucose transporters (Choeiri et al., 2005), and also in the case that the anatomy of the blood supply system provides much more glucose than is actually used in physiological conditions. Although some of the studies whose datasets we used showed that resting rates of glucose use are linearly correlated to capillary density across sites in the rat brain (Klein et al., 1986; Borowsky and Collins, 1989), which is consistent with supply-limitation of rates of glucose use at rest, we did not wish to assume that rates of glucose use at rest are determined by rates of blood flow through the capillaries in the local volume. To determine whether local capillary density correlates with local rates of blood supply, and whether the ratio between numbers of endothelial cells and the neurons they serve might determine how much blood, and therefore energy, is supplied per individual neuronal unit, we crossed our data on local densities of capillaries (the vascular fraction), endothelial, glial and neuronal cells with three published datasets on rates of blood flow in the resting rat brain (McCulloch et al., 1982; Nakai et al., 1983; Gjedde and Diemer, 1985; Figure 8). In each of these three datasets, local rates of glucose use are tightly, and linearly, correlated with local rates of blood flow measured in different brain structures (*r*^2^ = 0.874, *p* < 0.0001, and *r*^2^ = 0.578, *p* < 0.0001, respectively).

We find that rates of blood flow/min.100 g correlate positively and even more strongly than rates of glucose use with the capillary fractions and endothelial cell densities we measured in the same brain structures, such that structures with higher blood flow rates have proportionately higher capillary fraction (Figure 8A) and densities of endothelial cells (Figure 8B). Once more, we find that local rates of blood flow are not correlated with local densities of neurons or glial cells (Figures 8C,D).

The ratio between local rate of blood flow and local neuronal density indicates how much blood supply is available per neuronal unit per unit time. We find that this ratio is strongly linearly and inversely proportional to neuronal density (Figure 8E), like the rate of glucose use per neuronal unit (Figure 7E), with less blood supplied per neuronal unit in those locations where neuronal densities are higher. Consequently, as could be expected at this point, local variations in rate of blood flow per neuronal unit are strongly linearly correlated with the ratio of endothelial cells per neuron (Figure 8F), such that sites with more endothelial cells available per neuron also have higher rates of blood supplied per neuron.

Finally, to address the multiple correlations across the variables examined, we performed principal component analysis of the main variables available for each brain structure: local densities of endothelial cells, neurons, and glial cells; E/N and G/N ratios; rates of blood flow and glucose use; and rates of blood flow and glucose use per neuron. Strikingly, we find that rates of blood flow per neuron, glucose use per neuron, E/N and G/N ratios load very strongly in the first factor (factor loading, 0.943, 0.935, 0.952, and 0.965, respectively), together with glial cell density (factor loading, 0.599), accounting for 44.5% of the variance in the data. In the second factor, rates of blood flow and glucose use load together with endothelial cell density (factor loading, 0.993, 0.712, and 0.578, respectively), accounting cumulatively for 67.4% of variance in the data. Notably, local neuronal density does not load significantly in either factor, indicating that neuronal density is a truly independent variable in the dataset.

## Discussion

Here we find that, as we reported in the mouse (Ventura-Antunes and Herculano-Houzel, in review), capillary fraction and endothelial cell densities vary little across sites in the rat brain, compared to the highly variable neuronal cell densities across sites, with no consistent or universal correlation between capillary fraction (or endothelial cell densities) and neuronal or glial cell densities across those sites. Crucially, at the same time, we find that the small variations that do occur in local capillary fraction and endothelial cell density correlate strongly with independent measurements of local blood flow rate and glucose use at rest, while these measurements of local metabolic rates in the resting brain again bear no systematic relationship to local densities of neurons or glial cells. Most importantly, crossing our data on cellular distribution in the rat brain to published data on metabolism in matching brain sites revealed that the average resting rates of blood supply and glucose use per neuronal unit are strongly and linearly predicted by the anatomical ratio of endothelial cells per neuron across brain sites. Given that there is no other source of oxygen and glucose to the brain other than the capillary bed, it follows that local capillary density, which we confirm to correlate strongly with local rates of blood flow at rest, must be limiting to the rate at which energy (in the form of both oxygen and glucose, no matter the stoichiometry of their use) is *delivered* to brain sites, regardless of how many neurons are found at each location. Local capillary density must remain limiting to rates of energy *delivery* even in the case of variations in expression of glucose transporters, which necessarily occurs on top of limitations imposed by capillary density, for it is not possible to transport to neurons glucose that never arrived at a brain site because of limited capillary delivery. Moreover, since oxygen transport from hemoglobin to brain tissue depends exclusively on diffusion, and not on carriers or transporters, its availability is ultimately limited by the anatomy of the supply network – that is, by the density of the capillary bed. Thus, we propose that while variations in local capillary density are relatively minimal across sites in the rat brain, they are highly consequential to brain metabolism by determining the rate of energy supply to diverse numbers of neurons across locations in the brain. This proposition is fully developed into a supply-limited framework of brain metabolism in a companion paper (Herculano-Houzel and Rothman, 2022).

It is undisputed that sensory stimulation and other forms of focused mental activity increase local neuronal activity and brain metabolism (reviewed in Leithner and Royl (2014)), which in principle might appear to make our present anatomical study of questionable relevance to advancing our understanding of brain metabolism. Additionally, it could be argued that the capillary bed is plastic and can respond with proliferation to changes in activity. For example, dark-rearing and visual deprivation causes the visual cortex to develop with decreased vascularization (Toga, 1987; Argandoña and Lafuente, 1996), whereas complex visual experience leads to increased capillary density in the visual cortex (Sirevaag et al., 1988). We refute such likely criticism in two main ways. First, exactly because it is self-organized, the anatomy of the brain microvasculature at rest is a reflection of the history of brain activity (Black et al., 1987; Isaacs et al., 1992). For that reason, the resting, steady-state energy availability of the adult brain at rest, as well as its steady-state rate of energy use, is a reflection of its past history of activity, and represents an adequate supply. That past history reflected in the current distribution of capillaries captured in the images that we quantify includes any use-dependent modifications in local capillary density. Thus, understanding the distribution of capillaries in the brain provides unique insight into the self-organizing principles that govern brain metabolism at steady-state.

While we acknowledge that direct implications of our quantitative findings on the capillary distribution of fixed rat brains are currently limited to the resting (undisturbed awake) rat brain, the second argument for the relevance of our steady-state findings to the dynamically changing awake brain is our recent demonstration that local capillary densities are indeed a fundamental determinant of rates of brain metabolism also during sensory stimulation, even in the so-called “state of neurovascular uncoupling,” during which rates of blood flow increase much more than rates of oxygen use by brain tissue (Herculano-Houzel and Rothman, 2022, see companion paper). Increases in local rates of blood flow in the stimulated brain, presumably driven by variations in neuronal activity, are due to local changes in arterial dilation and increases in blood pressure, without capillary recruitment (Kuschinsky and Paulson, 1992; Jesperson and Ostergaard, 2012; Leithner and Royl, 2014). The increased local rate of blood flow would lead to a proportionally increased rate of oxygen transfer from blood to brain tissue if that rate weren’t already near maximal, constrained by the combination of capillary surface area (and thus capillary density), hemoglobin oxygenation (which is typically already saturated at rest, at 100% of arterial values), and hematocrit (which, on this time scale, is unchanging). Because there is no effective capillary recruitment in the awake brain, the increase in rate of oxygen use in the stimulated brain is only modest, and no longer linearly coupled to the increase rate of blood flow – but, importantly, the increased rate of oxygen use remains fully predictable by local capillary density (Herculano-Houzel and Rothman, 2022).

One of the strengths of our approach is that it allows the establishment of parallels between local capillary density (and thus local rate of blood flow) and local numbers of neurons and glial cells. While we did not measure actual rates of blood delivery per neuron, our finding that the ratio of endothelial cells per neuron is a strong linear predictor of average rate of blood flow per neuronal unit allows us to predict the implications to brain physiology, just like the capillary-to-muscle fiber ratio of skeletal muscle is regarded as a critical determinant of muscle metabolic capacity (Hepple et al., 1985; Wagner, 2000). We find that the economy of the working brain balances sites where as many as 60 neurons share blood supplied by a single endothelial cell, such as the granular layer of the cerebellum; sites where only two neurons (as in the thalamus and midbrain) or three neurons (as in the non-hippocampal pallium) share a single endothelial cell’s worth of blood supply; sites where there is about one endothelial cell for every neuron (as in the hindbrain); and sites where endothelial cells exceed the number of neuronal cell bodies consuming the energy they supply (as in the molecular layers of the hippocampus and cerebellum). With the much smaller variation in endothelial cell density (and therefore capillary density) than in neuronal density, it appears that the particular density at which neurons occur at different brain sites determines the economy of energy distribution amongst neighboring neurons, with more competition for energy in those sites where more neurons share the limited resources transported in the blood.

It is possible that some of the variation we find in cell densities and capillary fraction are due to unequal shrinkage of different brain structures during fixation and immunohistochemical processing. However, in the event of enough differential shrinkage across sites to affect the correlations that we report, we would expect that densities of both neurons and endothelial cells would be affected similarly at each site, which would artificially increase the chances that we find strong positive correlations between these two variables across sites. Our main finding of no strong or consistent correlation between neuronal and endothelial cell densities is thus the opposite of what one would expect if variable tissue shrinkage were a significant issue in our dataset.

By showing that variations in local capillary densities are at the same time very limited but also directly and linearly correlated to local rates of blood flow and glucose use at rest, our study raises an important new issue of what determines local capillary densities in the developing and in the steady-state adult brain. Activity-dependent formation of new capillaries is a possibility (Toga, 1987; Sirevaag et al., 1988; Argandoña and Lafuente, 1996), presumably leading to higher capillary densities in sites of higher neuronal activity, especially during development. However, the lack of correlation between local neuronal density and capillary density in the rat (this study) and mouse brain (Ventura-Antunes and Herculano-Houzel, in review) as well as between local density of synapses and capillary density in the mouse (Ventura-Antunes and Herculano-Houzel, in review), when the rate of energy use by neurons is believed to be heavily dependent on synaptic activity (Attwell and Laughlin, 2001), puts into question the extent to which local differences in intrinsic or stimulus-driven neuronal activity might drive variations in local capillary density. Moreover, the recent observation that endothelial cells in the adult mouse brain are as old as the neurons that they feed (Arrojo e Drigo et al., 2019) raises doubts regarding the magnitude of the impact of activity-dependent changes in capillary density in adult animals. On the other hand, the physics of scaling of distribution networks predicts that capillary densities should be determined by the size of the system (Banavar et al., 2002), which is consistent with the small degree of variation that we report here. It will be interesting to pursue the question of what determines the small but consequential variations in local capillary densities in the brain that we report here.

An important incidental finding of the present study is that endothelial cells and glial cells appear to vary together in numbers and density across gray matter structures, with a somewhat constant ratio of about three glial cells to every endothelial cell in these structures. Astrocytic endfeet are closely associated with endothelial cells, and astrocytes play a key role in providing neurons with energy substrates either directly removed or transformed from substances transferred from the blood (Magistretti and Pellerin, 1996). Given that astrocytes are only about 5–10% of non-neuronal cells of the rodent brain (Herculano-Houzel et al., 2006; Sun et al., 2017), our finding of an average of ca. 3 glial cells per endothelial cell suggests that there is at most one astrocyte per endothelial cell in the different gray matter structures in the rat brain, even though non-quantitative microscopy indicates, from the early studies of Ramón y Cajal (1995), that individual capillaries are covered by multiple astrocytic endfeet. Combined with the realization that astrocytes are actually a minority of glial cells (Sun et al., 2017), our finding of ca. 3 glial cells per endothelial cell suggests that the various endfeet that cover each endothelial cells very likely originate from the same individual astrocyte, and at best the few neighboring astrocytes that tile that territory, such that, overall, each astrocyte contacts at most a couple of endothelial cells simultaneously. It will be interesting to investigate directly this quantitative relationship in the future. Until then, the small and at best 1:1 ratio of astrocytes to endothelial cells that we suggest points to yet another way in which brain metabolism is constrained by supply, given that glial cells, like endothelial cells, occur at a narrow range of densities, and therefore the number of neurons served by every astrocyte depends simply on how many neurons occur within the territory of an individual astrocyte (Herculano-Houzel, 2014). Thus, we propose that neuronal metabolism is limited not only in how much blood is supplied to their territory by capillaries, but also by how many other neurons share their astrocytic support.

It follows that those brain areas with higher neuronal densities (because these are not compensated by increased capillary density) should be particularly susceptible to alterations that disrupt blood supply and astrocyte-supported metabolism, such as ischemia, metabolic diseases, and aging. We thus predict that brain structures with lower E/N ratios, and not simply those brain areas with highest metabolic rates, should be the most vulnerable and the first parts of the brain to be afflicted once blood supply is compromised. For instance, the entorhinal cortex, one of the first cortical areas to be affected in Alzheimer’s disease, is one such area of fairly low metabolic rate, but with its high neuronal density, it actually has the lowest E/N ratio of the non-hippocampal pallium (0.024, or 42 neurons to each endothelial cell), followed by the hippocampal dentate gyrus (0.126, or 8 neurons to each endothelial cell; Table 3), another highly vulnerable structure. Consistently with our proposition that neuronal competition for a limited capillary supply is a determinant factor in brain health and disease, it is now increasingly recognized that cerebrovascular pathology contributes to dementia and clinically diagnosed Alzheimer’s disease (Arvanitakis et al., 2016), and blood flow reductions that impair the hemodynamic responses are increasingly recognized in normal aging (Brown and Thore, 2011) as well as in the early stages of Alzheimer’s disease (Sweeney et al., 2018).

**TABLE 3:** Average rates of glucose use/neuron and blood flow/neuron in different structures of the rat brain.

Structure	*n*	Capillary fraction,%	Endothelial cells/ mm^2^	Rate of blood flow	Rate of glucose use	Neurons/ mm^2^	Glial cells/ mm^2^	E/N	Glucose use/N	Blood flow/N
Amygdala	12	2.8 ± 0.6	36,707 ± 4,789	89.5 ± 8.2	47.5 ± 5.4	164,784 ± 40,877	76,391 ± 15,818	0.237 ± 0.071	0.443 ± 0.199	0.000579 ± 0.000174
Auditory cx	12	3.7 ± 0.5	43,413 ± 5,605	182.5 ± 47.7	123.3 ± 33.5	137,864 ± 2,394	68,384 ± 8,936	0.315 ± 0.036	0.613 ± 0.221	0.001326 ± 0.000347
Cerebellar cx	15	3.2 ± 0.2	48,278 ± 8,177	109.5 ± 0.5	51.4 ± 11.6	1,041,381 ± 69,194	112,677 ± 19,181	0.047 ± 0.010	0.328 ± 0.107	0.000106 ± 0.000007
Cerebellar dentate nucleus	3	5.8 ± 0.6	98,379 ± 9,658	168	81	64,748 ± 21.673	250,627 ± 50,427	1.651 ± 0.591	3.933 ± 1.120	0.002837 ± 0.001097
Other cerebellar nuclei	6	5.3 ± 0.8	70,718 ± 13.423	165	103 ± 3.3	52,962 ± 9,768	189,798 ± 24,645	1.365 ± 0.296	4.739 ± 0.968	0.003210 ± 0.000695
Cerebellar white matter	12	2.0 ± 0.2	26,530 ± 1,822	44	36	7,770 ± 5,910	198,945 ± 33,998	2.218 ± 0.233	31.689 ± 16.851	0.003853 ± 0.000775
Cochlear nucleus	9	4.8 ± 0.5	72,236 ± 13,841	187.5 ± 19.2	96.7 ± 39.5	169,681 ± 17,271	216,827 ± 62,126	0.435 ± 0.113	1.701 ± 0.732	0.001115 ± 0.000159
Colliculi, inferior	21	4.5 ± 0.7	60,763 ± 6,784	220.5 ± 39.4	132.4 ± 69.8	166,205 ± 48,246	144,593 ± 16,879	0.393 ± 0.117	0.893 ± 0.642	0.001493 ± 0.000449
Colliculi, superior	18	4.5 ± 0.8	66,135 ± 12,822	133.5 ± 8.2	80.3 ± 14.2	220,438 ± 51,824	143,051 ± 20,241	0.331 ± 0.145	1.130 ± 0.418	0.000646 ± 0.000196
Corpus callosum	15	1.2 ± 0.1	19,178 ± 4,198	53.7 ± 11.1	34.0 ± 8.0	789 ± 344	335,467 ± 38,808	32.652 ± 22.707	160.040 ± 119.914	0.086838 ± 0.082989
Cx, cingulate	6	4.1 ± 0.5	49,928 ± 5,797	113	77.5 ± 47.7	156,932 ± 25,283	86,253 ± 16,410	0.321 ± 0.032	0.379 ± 0.238	0.000735 ± 0.000120
Cx, entorhinal	6	2.8 ± 0.6	37,230 ± 7,831	102	36.5 ± 10.4	127,166 ± 18,215	82,368 ± 10,110	0.291 ± 0.019	0.240 ± 0.093	0.000815 ± 0.000124
Cx, frontal	15	3.3 ± 0.1	49,274 ± 4,569	127 ± 17.9	73.8 ± 26.2	113,670 ± 1,780	67,123 ± 3,886	0.434 ± 0.041	0.371 ± 0.146	0.001118 ± 0.000159
Cx, infralimbic	2	3.1 ± 1.3	36,650 ± 7,127	n.a.	71	123,446 ± 11,467	81,417 ± 8,122	0.295 ± 0.030	0.490 ± 0.139	n.a.
Cx, insular	3	3.1 ± 1.1	37,198 ± 6,208	n.a.	71	127,922 ± 21,383	65,195 ± 13,427	0.298 ± 0.006	0.344 ± 0.073	n.a.
Cx, occipital	3	3.4 ± 0.8	46,230 ± 11,423	n.a.	100	170,343 ± 32,615	77,017 ± 14,904	0.270 ± 0.017	0.477 ± 0.160	n.a.
Cx, parietal	12	3.6 ± 0.7	49,533 ± 5,962	108.5 ± 9.3	76.5 ± 29.4	183,626 ± 15,967	78,584 ± 7,160	0.269 ± 0.015	0.313 ± 0.125	0.000595 ± 0.000074
Cx, posterior parietal	3	3.3 ± 0.7	43,987 ± 7,221	152	101	162,350 ± 24,445	73,894 ± 16,293	0.271 ± 0.022	0.477 ± 0.185	0.000951 ± 0.000151
Cx, prefrontal	3	3.2 ± 0.3	49,561 ± 8,556	201	130	112,567 ± 1,727	61,276 ± 11,427	0.440 ± 0.072	0.647 ± 0.110	0.001786 ± 0.000027
Cx, sensorimotor	6	3.6 ± 0.8	46,983 ± 3,496	158	111.0 ± 7.7	122,069 ± 14,425	78,440 ± 10,460	0.387 ± 0.020	0.417 ± 0.108	0.001309 ± 0.000161
Cx, temporal	3	3.2 ± 0.5	39,511 ± 8,956	n.a.	122	152,321 ± 16,716	54,205 ± 5,755	0.258 ± 0.036	0.736 ± 0.242	n.a.
Cx, visual	15	3.8 ± 0.8	49,924 ± 4,283	119.3 ± 25.4	77.0 ± 33.0	191,932 ± 19,894	77,985 ± 8,085	0.261 ± 0.017	0.285 ± 0.137	0.000629 ± 0.000153
Cx, white matter	2	1.5 ± 0.3	23,472 ± 2,299	n.a.	n.a.	4,811 ± 1,748	317,203 ± 49,088	5.317 ± 2.409	n.a.	n.a.
Globus pallidus	9	2.1 ± 0.3	26,476 ± 3,017	72.0 ± 4.4	46.7 ± 4.0	20,346 ± 4,964	186,124 ± 44,030	1.413 ± 0.525	4.717 ± 3.236	0.003744 ± 0.001019
Habenula, lateral	4	6.0 ± 3.1	54,879 ± 13,102	168	95.5 ± 7.1	160,722 ± 19,900	185,854 ± 36,176	0.353 ± 0.125	4.187 ± 3.672	0.001057 ± 0.000160
Hippocampus, dentate gyrus	9	2.9 ± 0.6	35,300 ± 5,459	165	52.7 ± 16.0	306,151 ± 86,386	78,247 ± 11,721	0.126 ± 0.004	1.323 ± 0.519	0.000589 ± 0.000230
Hippocampus, DG, molecular layer	9	2.9 ± 0.6	34,669 ± 3,279	n.a.	83	16,309 ± 9,217	81,551 ± 11,540	3.562 ± 2.877	43.802 ± 44.689	n.a.
Hippocampus, total	15	2.3 ± 0.5	32,156 ± 2,064	103.5 ± 8.2	64.3 ± 11.5	204,106 ± 142,561	62,242 ± 8,536	0.324 ± 0.288	2.093 ± 0.740	0.001003 ± 0.000918
Hypothalamus, anterior	6	3.1 ± 0.7	41,958 ± 10,233	n.a.	47.0 ± 9.9	142,472 ± 34,001	127,492 ± 15,818	0.297 ± 0.046	0.238 ± 0.095	n.a.
Hypothalamus, medial	3	2.7 ± 0.5	31,728 ± 6,849	n.a.	42	105,574 ± 18,062	116,939 ± 24,220	0.300 ± 0.041	0.286 ± 0.094	n.a.
Hypothalamus, posterior	3	3.8 ± 0.7	46,098 ± 6,761	n.a.	56	139,088 ± 37,729	129,347 ± 54,348	0.357 ± 0.142	0.330 ± 0.147	n.a.
Hypothalamus, total	12	3.1 ± 0.3	38,440 ± 4,584	112.0 ± 20.0	46.0 ± 15.2	127,544 ± 2,723	118,684 ± 19,456	0.301 ± 0.032	0.270 ± 0.107	0.000879 ± 0.000158
Internal capsule	6	1.7 ± 0.3	24,782 ± 2,169	50	33.5 ± 1.6	1,871 ± 1,271	205,201 ± 28,106	21.273 ± 16.150	56.308 ± 56.501	0.040964 ± 0.031097
Nucleus accumbens, whole	6	2.2 ± 0.2	30,458 ± 1,383	118.0 ± 12.0	54.0 ± 28.5	165,228 ± 41,251	117,927 ± 25,896	0.194 ± 0.048	0.448 ± 0.279	0.000752 ± 0.000198
Nucleus accumbens, core	6	2.3 ± 0.3	30,145 ± 2,708	n.a.	73.5 ± 7.1	165,678 ± 41,847	110,471 ± 31,364	0.189 ± 0.032	0.502 ± 0.167	n.a.
Nucleus accumbens, shell	3	2.1 ± 0.2	30,528 ± 3,546	n.a.	65	164,402 ± 45,654	123,813 ± 29,531	0.198 ± 0.069	0.679 ± 0.337	n.a.
Pontine nuclei	9	4.5 ± 1.2	66,259 ± 12,780	134.0 ± 11.0	60.0 ± 9.9	122,441 ± 10,036	160,126 ± 5,871	0.537 ± 0.061	1.385 ± 0.577	0.001101 ± 0.000129
Olive, inferior	3	5.9 ± 1.7	92,768 ± 23,940	177	64	88,744 ± 46,150	170,207 ± 22,130	1.207 ± 0.467	3.888 ± 1.966	0.002616 ± 0.001823
Olive, superior	9	6.2 ± 1.1	88,952 ± 12,401	204	128.3 ± 14.1	55,186 ± 21,728	212,768 ± 14,030	1.997 ± 1.130	5.169 ± 1.626	0.004355 ± 0.002244
Reticular nuclei, gigantocellular	3	3.3 ± 0.4	39,007 ± 3,601	103	47	36,949 ± 6,592	119,824 ± 14,483	1.083 ± 0.247	1.685 ± 0.843	0.002855 ± 0.000568
Retinular nuclei, parvocellular	3	3.5 ± 0.5	42,782 ± 4,743	119	50	52,369 ± 21,678	120,549 ± 16,571	0.920 ± 0.372	1.351 ± 0.393	0.002656 ± 0.001400
Striatum	15	3.1 ± 0.5	39,629 ± 3,471	128.5 ± 3.8	91.3 ± 15.9	121,870 ± 10,383	88,332 ± 15,483	0.325 ± 0.008	0.358 ± 0.105	0.001061 ± 0.000098
Substantia nigra, pars compacta	1	4.1	45,215	n.a.	65.0 ± 6.6	75,358	113,037	0.600	6.500 ± 0.849	n.a.
Substantia nigra, pars reticulata	9	4.1 ± 1.6	53,496 ± 18,625	86	48.5 ± 4.9	57,631 ± 22,061	129,219 ± 18,830	0.966 ± 0.243	1.439 ± 0.225	0.001688 ± 0.000681
Substantia nigra, total	1	3.0	36,675	93	52	51,163	114,980	0.717	2.006	0.001818
Thalamus	14	2.9 ± 0.2	37,240 ± 3,487	135.5	82.8 ± 20.0	69,226 ± 5,826	105,875 ± 8,675	0.538 ± 0.018	0.492 ± 0.118	0.001954 ± 0.000191
Vestibular nucleus	15	4.7 ± 0.5	55,166 ± 8,949	189.3 ± 11.9	95.0 ± 35.8	43,317 ± 17,927	149,341 ± 10,858	1.538 ± 0.791	3.389 ± 2.053	0.005078 ± 0.001965

*Values correspond to the mean ± SD across the five datasets on rates of glucose use [in mmol/(100 g.min); Sokoloff et al., 1977; McCulloch et al., 1982; Nakai et al., 1983; Gjedde and Diemer, 1985; Levant and Pazdernik, 2004] and two datasets on blood flow rates [in ml/(100g.min); McCulloch et al., 1982; Nakai et al., 1983], obtained by dividing reported local rates by the average neuronal density in the same structures in each of three rats. N indicates the number of sites used to determine cell densities for each structure, also provided in the table, along with capillary fraction.*

Recognizing that the relative ratio of local neuronal densities to local capillary density is of such potentially fundamental relevance to brain health raises new questions about how local capillary density is determined, which is complicated by the fact that our understanding of the source of variations in local neuronal size that lead to variations in neuronal density across brain sites remains lacking. During the early stages of development, the radial glial cells that give rise to early neurons and form migrating routes for these newly generated cells (Rakic, 1972) also guide the formation and stabilization of a primitive vascular network (Ma et al., 2013). It is likely that multiple factors influence the formation of what will ultimately be the capillary bed of the brain, but our companion study in the mouse indicates that synaptic density is not one of them (see text footnote 1). We plan to study the parallel development of the distribution of capillaries and neurons in different mammalian species to shed light on the sources of variation in capillary density, and in particular on the origin of the higher capillary densities in hindbrain structures reported here. One testable hypothesis to be examined is that the accentuated growth of neuronal arbors that occurs in the cerebral cortex during neuronal differentiation pushes already formed capillaries away from one another, thus leading to lower capillary densities in the cerebral cortex than those observed in the hindbrain. Another possibility is that there are fundamental differences between the anatomy and physiology of the vascularization of the cerebrum and of the hindbrain, which indeed branch off from two different main arteries: the internal carotid and the basilar artery. It will be interesting to investigate how that difference could also account for the higher capillary densities that we report in territories vascularized by the basilar artery.

Finally, our observations strongly indicate that local capillary fraction and/or endothelial cell density in the rat brain serve as accurate proxies for local metabolic rate, especially blood flow rate, across brain structures. Because local capillary fraction and endothelial cell densities (which we find to be linearly correlated to each other) are readily quantifiable in thin 2D sections of fixed brains, they show great promise in serving as proxies for studies of how brain metabolism scales across species. To that end, it will be necessary to determine whether the same relationship between capillary density and rate of blood flow at rest is shared across mammalian species. However, together with the observation that the capillary bed is the most restrictive portion of the circulatory system to blood flow (Gould et al., 2017), the finding that resting brain metabolic rate scales consistently across mammalian species (Herculano-Houzel, 2011) strongly suggests that the relationship between capillary density and rate of blood flow is shared universally across mammalian species. In the case that such a universal relationship is confirmed, we will finally be able to extend studies of brain metabolism to a wide range of mammalian species that would be impossible to examine alive, but whose fixed brains are approachable.

## Data Availability Statement

The original contributions presented in this study are included in the article/supplementary material, further inquiries can be directed to the corresponding author/s.

## Ethics Statement

Ethical review and approval was not required for the animal study because brains were purchased from BrainBits already perfused.

## Author Contributions

SH-H conceived the study. LV-A and OD executed all data acquisition. All authors analyzed the data. SH-H and LV-A wrote the manuscript.

## Conflict of Interest

The authors declare that the research was conducted in the absence of any commercial or financial relationships that could be construed as a potential conflict of interest.

## Publisher’s Note

All claims expressed in this article are solely those of the authors and do not necessarily represent those of their affiliated organizations, or those of the publisher, the editors and the reviewers. Any product that may be evaluated in this article, or claim that may be made by its manufacturer, is not guaranteed or endorsed by the publisher.

## Footnotes

1 Ventura-Antunes, L., and Herculano-Houzel, S. (2022). Energy supply per neuron is constrained by capillary density in the mouse brain. *Front. Integr. Neurosci.*
